# Untangling Dual-Targeting Therapeutic Mechanism of Epidermal Growth Factor Receptor (EGFR) Based on Reversed Allosteric Communication

**DOI:** 10.3390/pharmaceutics13050747

**Published:** 2021-05-18

**Authors:** Yuran Qiu, Xiaolan Yin, Xinyi Li, Yuanhao Wang, Qiang Fu, Renhua Huang, Shaoyong Lu

**Affiliations:** 1 Key Laboratory of Cell Differentiation and Apoptosis of Chinese Ministry of Education, Department of Pathophysiology, School of Medicine, Shanghai Jiao Tong University, Shanghai 200025, China; qiuyuran.georgina@sjtu.edu.cn (Y.Q.); Ashley_Li@sjtu.edu.cn (X.L.); wangyh2000@sjtu.edu.cn (Y.W.); 2 Department of Radiotherapy, Changhai Hospital (Hongkou District), Naval Medical University, Shanghai 200081, China; yxlorchid@163.com; 3 Department of Orthopedics, Shanghai General Hospital, School of Medicine, Shanghai Jiao Tong University, Shanghai 200080, China; 4 Department of Radiation, Renji Hospital, School of Medicine, Shanghai Jiao Tong University, Shanghai 200120, China

**Keywords:** epidermal growth factor receptor, dual-targeting therapeutics, allosteric regulation, molecular dynamics simulations, allosteric site

## Abstract

Dual-targeting therapeutics by coadministration of allosteric and orthosteric drugs is drawing increased attention as a revolutionary strategy for overcoming the drug-resistance problems. It was further observed that the occupation of orthosteric sites by therapeutics agents has the potential to enhance allosteric ligand binding, which leads to improved potency of allosteric drugs. Epidermal growth factor receptor (EGFR), as one of the most critical anti-cancer targets belonging to the receptor tyrosine kinase family, represents a quintessential example. It was revealed that osimertinib, an ATP-competitive covalent EGFR inhibitor, remarkably enhanced the affinity of a recently developed allosteric inhibitor JBJ-04-125-02 for EGFR^L858R/T790M^. Here, we utilized extensive large-scale molecular dynamics simulations and the reversed allosteric communication to untangle the detailed molecular underpinning, in which occupation of osimertinib at the orthosteric site altered the overall conformational ensemble of EGFR mutant and reshaped the allosteric site via long-distance signaling. A unique intermediate state resembling the active conformation was identified, which was further stabilized by osimertinib loading. Based on the allosteric communication pathway, we predicted a novel allosteric site positioned around K867, E868, H893, and K960 within the intermediate state. Its correlation with the orthosteric site was validated by both structural and energetic analysis, and its low sequence conservation indicated the potential for selective targeting across the human kinome. Together, these findings not only provided a mechanistic basis for future clinical application of the dual-targeting therapeutics, but also explored an innovative perception of allosteric inhibition of tyrosine kinase signaling.

## 1. Introduction

Allostery or allosteric regulation, where protein orthosteric sites are fine-tuned by topographically distal allosteric sites, orchestrates a plethora of biological processes such as enzyme catalysis, cellular metabolism, transcriptional regulation, and signal transduction, and is thus considered as “the second secret of life” [1,2,3]. As one of the most efficient and precise paradigms to tweak proteins’ functional activity and an important supplement to the traditional orthosteric-targeting strategy, allosteric modulators by targeting allosteric sites have enhanced specificity and reduced adverse effects, thereby presenting a promising avenue for modern drug development [4,5,6]. The latest decade has witnessed the upsurge of structural biology and protein allostery research, which led to inspiring success in allosteric drug discovery throughout an extended list of critical therapeutic targets such as kinases [7,8,9], Ras [10,11,12,13,14], and G-protein-coupled receptors [15,16], proving the enormous potential of allosteric regulation.

In addition to single targeting at either orthosteric or allosteric sites, the dual-targeting therapeutics at both sites have recently gained increasing recognition for its favorable performances [17,18]. Several studies have validated that coadministration of allosteric drugs can restore the efficacy of orthosteric drugs by precisely modulating the orthosteric sites for ligand accommodation, thus conquering the notorious problem of drug resistance. For example, the combination of allosteric inhibitor ABL001 and classical tyrosine kinase inhibitors (TKIs) targeting BCR-ABL, including imatinib, nilotinib, and dasatinib, in the treatment of chronic myeloid leukemia (CML) yielded a durable and complete regression of the malignancy, as well as tackled the recalcitrant problem of drug-resistance [19]. A clinical trial (Clinical Trial Number: NCT02081378i) has been launched to evaluate the therapeutic effects of combining ABL001 with TKIs for treating CML. Therefore, the dual-targeting therapeutics has been established as a revolutionary strategy to circumvent drug resistance. Notably, in addition to the improvement of orthosteric drug-resistance by allosteric modulators, another fascinating phenomenon has also been observed in multiple cases, in which the presence of orthosteric ligands dramatically contributes to the affinity of allosteric ligands in reverse [20,21,22]. However, the underlying mechanism remains unclarified.

To address this challenge, we make recourse to the reversed allosteric communication theory, which serves as a supplement to the classical allosteric communication theory. According to this recently proposed theory, the communication signals can propagate not only from the allosteric site to the orthosteric site, but also reversely from the orthosteric site to the allosteric site, leading to a close bi-direction correlation between the two sites [23,24,25,26]. This theory has been validated in several studies as examined with a series of classical allosteric proteins, including 15-lipoxygenase [27] and protein kinase 1 [23], and further promoted the discovery of several novel allosteric sites. Recently, based on the reversed allosteric communication, we have developed a combined computational and experimental strategy to discover cryptic allosteric sites of sirtuin 6 (SIRT6), providing a starting point for SIRT6 allosteric drug design [28]. Herein, we hypothesized that the altered efficacy of allosteric inhibitors observed in experiments was attributed to reversed allosteric communication, whereby the conformation of allosteric sites can be shifted by perturbations at the functional sites, contributing to the emergence or stabilization of allosteric pockets. To test our hypothesis, the epidermal growth factor receptor (EGFR), one of the most-studied anti-cancer targets in the era of modern medicine, was chosen for further investigation in this study.

EGFR, a member of the receptor tyrosine kinases family, represents a pivotal component in the network of signal transduction, cell differentiation, proliferation, and survival [29]. While accumulating evidence has shown that the malfunctions of EGFR caused by activating mutations initiate constitutive engagement and activation of downstream effector signaling, which account for approximately 10~30% oncogenesis of non-small cell lung cancer (NSCLC) [30,31]. Thus far, oncogenic EGFR mutants portray an intriguing therapeutic target for anti-cancer treatment.

**Figure 1:**
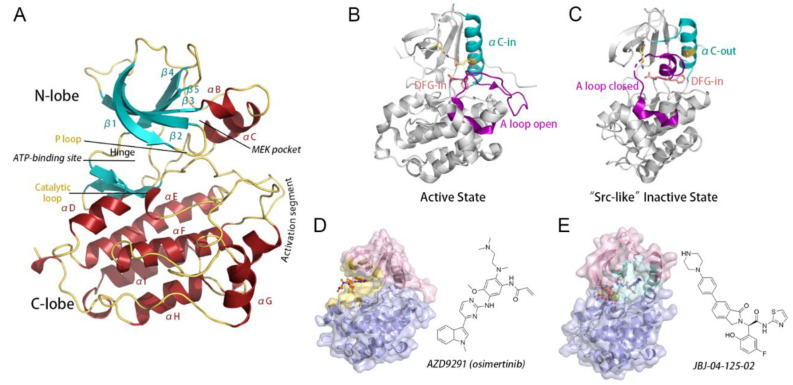
Structure overview of EGFR and its inhibitors. (**A**) Structure of EGFR with major secondary structural elements and functional domains specified. (**B**) The active state structure (αC-in, A loop open, DFG-in) of EGFR CD (PDB ID: 2GS2). Key structural elements are colored in cyan (αC helix) and purple (A loop). Residues K745 and E762 are highlighted as yellow sticks with salt bridge depicted by orange dashed lines, while the DFG motif is highlighted as salmon sticks. (**C**) The “Src-like” inactive state structure (αC-out, A loop closed, DFG-in) of EGFR CD (PDB ID: 2GS7). (**D**) Binding mode and chemical structure of osimertinib. (**E**) Binding mode and chemical structure of JBJ-04-125-02.

Structurally, EGFR is composed of an extracellular receptor, a transmembrane region, and an intracellular catalytic domain (CD). The activation of CD is induced by the binding of EGF on the cell-surface receptor, which triggers the dimerization of EGFR and the following transduction of downstream signaling. CD contains a smaller N-lobe, which is formed by five β-strands and the αC-helix, and a mainly helical C-lobe that embraces a highly flexible activation loop (A loop) (Figure 1A). A deep cleft at the interface between the two lobes serves as a binding pocket for the adenine ring of ATP. The key to determining whether CD adopts an activation or inactivation state includes the conformation of three conserved structural elements: the Asp-Phe-Gly (DFG) motif, the αC-helix, and the A loop. In the active state of EGFR, the catalytically important residue D855 points inside the ATP-binding site to stabilize the ATP-loaded EGFR complex (“DFG-in”), and the αC-helix adopts a so-called “αC-in” conformation, which is locked adjacent to the ATP-binding site via a vital K745-E762 salt bridge between the β3 strand and the αC-helix (Figure 1B). Meanwhile, the A loop maintains a β9 strand and settles on an extended “A loop open” conformation compatible with substrate docking. While any conformational deviation from this structure leads to functional inactivation of EGFR. The “Src-like” inactive conformation is one of the most classic catalytically inactive states, which is characterized by the formation of two short α-helices in the A loop and its interaction with the out-rotated αC-helix (Figure 1C).

Multiple somatic mutations at the CD have clinical significance due to their activating or drug-resistant effects, among which L858R is the most common oncogenic mutation occurring in EGFR and is frequently detected in a large subset of patients with NSCLC [31,32,33]. By targeting the orthosteric ATP-binding site in the interlobar cleft, TKIs compete with ATP and thereby blunt aberrant EGFR activation caused by oncogenic mutations such as L858R, which has revolutionized the treatment of NSCLC. Until recently, 1st and 2nd generations of reversible TKIs, including afatinib, gefitinib, and erlotinib, have been established as the standard initial therapy for NSCLC patients laboring EGFR mutations [34,35,36]. However, secondary drug-resistance mutation T790M occurs within ~60% of treated patients, which enhances EGFR affinity for ATP and thus diminishes the potency of reversible TKIs [37,38]. Mutant-selective irreversible TKIs, including the FDA-approved osimertinib (AZD9291), form a covalent bond with C797 at the edge of the ATP site to outcompete ATP in EGFR^L858R/T790M^ mutants (Figure 1D) [39,40]. However, the tertiary C797S mutation dramatically hampers the efficacy of osimertinib by 100 to 1,000-fold in EGFR phosphorylation and oncogenesis inhibition, posing a challenge to its clinical performance [39,41]. Hence, with such reality check, an effective anti-resistance strategy may represent the ultimate answer to EGFR-mutated NSCLC.

To circumvent acquired drug-resistance against TKIs, a set of allosteric inhibitors targeting EGFR were previously introduced, including EAI001, EAI045, and JBJ-04-125-02 (shortened as “JBJ” in the article) (Figure 1E) [20,42,43,44]. To our knowledge, all current allosteric EGFR inhibitors target the MEK-pocket, which is generated by the outward displacement of the αC-helix and was first identified in mitogen-activated protein (MAP) kinase kinases (MEKs) (Figure 1E) [45]. Importantly, JBJ was reported to have synergistic effects together with osimertinib during coadministration [20]. The combination of JBJ and osimertinib led to not only an evident delay in orthosteric drug-resistance, but also a unique enhancement of JBJ affinity in EGFR^L858R/T790M^ in the presence of osimertinib. Such enhancement is independent of the amount of available EGFR monomers present in the cells, which leads to enhanced efficacy of the combination. Consequently, the dual-targeting therapeutics in EGFR proposed a solution to overcome resistance and meanwhile improve therapeutic outcomes. These findings highlighted EGFR as an ideally allosteric protein and a potential target for dual-targeting therapeutics.

In this study, large-scale atomistic molecular dynamics (MD) simulations revealed that the dynamic landscape of EGFR^L858R/T790M^ was markedly reshaped upon the binding of osimertinib, characterized by the stabilization of a catalytically inactive intermediate substrate that is first reported in EGFR, thus modulating the allosteric MEK-pocket. Moreover, we described the stepwise transition pathway of EGFR approaching the “Src-like” inactive states, as well as the community network of allosteric signaling, in an effort to gain an atomistic understanding of the reversed allosteric regulatory mechanism within the CD. Importantly, based on the reversed allosteric communication, we identified a potential druggable allosteric pocket (X-pocket) presented in osimertinib-loaded EGFR^L858R/T790M^, which was well exposed and can be readily targeted in both the active and intermediate states of EGFR. The allosteric crosstalk between the X-pocket and the ATP-binding site was analyzed using structural and energetic analysis. Moreover, the nonconservation characteristics of the X-pocket across the human kinome confirmed its potential to yield selective inhibitors targeting EGFR. Our study revealed a dual-targeting therapeutic mechanism that allosteric sites were highly correlated with orthosteric drugging via reversed allosteric communication, which provided a mechanistic basis for future clinical application of the dual-targeting therapeutics. Furthermore, the verification methodology utilized in this study led to the discovery of a novel, potential allosteric pocket. Our findings may aid future rational design of a new generation of selective EGFR inhibitors and provide an innovative perception of the modulation of receptor tyrosine kinase family.

## 2. Materials and Methods

### 2.1. Construction of Stimulated Systems

Three systems were constructed and subjected to MD simulations, including the *apo* EGFR^L858R/T790M^, EGFR^L858R/T790M^–osimertinib, and EGFR^L858R/T790M^–osimertinib–JBJ. The construction of the EGFR^L858R/T790M^–osimertinib complex was based on a co-crystal structure of human EGFR^L858R/T790M^ monomeric form from the RCSB Protein Data Bank (PDB ID: 4I1Z) [46], while the EGFR^L858R/T790M^–osimertinib–JBJ stemmed from a co-crystal structure of human EGFR in complex with JBJ and ANP (PDB ID: 6DUK) [20]. The remodel of the missing residues, as well as the mutations of L858 and T790, was conducted with Discovery Studio, and osimertinib was docked into the ATP-binding site using a covalent docking protocol. The structure of free EGFR^L858R/T790M^ was extracted from the EGFR^L858R/T790M^–osimertinib complex. The water molecules in the crystal structures were removed.

### 2.2. Covalent Docking

The docking of osimertinib was performed by Maestro Advantage Schrödinger (Maestro, Schrödinger, LLC, New York, NY, USA). The structures of EGFR^L858R/T790M^, EGFR–JBJ–ADP, and osimertinib were separately loaded and prepared in Maestro. The centroid of ADP was selected as the center for grid generation with a site size of 20 × 20 × 20 Å^3^. Constraints of the sulfhydryl group at C797 were specified as the electron donor for Michael Addition during grid generation. The generated grid file and the prepared small ligand were used as input files to perform covalent docking. The 50 top-ranked poses of osimertinib were extracted and analyzed for rationality. Finally, the lowest energy-docking pose was utilized for further simulations.

### 2.3. MD Simulations

The C797 residue bonded with osimertinib was viewed as a single modified amino acid, and the Amber ff14SB force field and general amber force field (GAFF) were employed to prepare the parameter files for minimizations and simulations [47]. All three complexes were solvated in an orthorhombic TIP3P water box and then the counterions were added for neutralization [48]. To mimic the physical condition inside human cells, 0.15 mol L^−1^ NaCl was further solvated into each system.

Each system was subjected to two rounds of energy minimization. They underwent 2000 steepest descent and 3000 conjugate gradient minimization steps with a backbone positional restraint of 500 kcal (mol^−1^ Å^−2^) for 10 ps, followed by another round of 4000 steepest descent and 6000 conjugate gradient minimization steps without any constraint for 20 ps. After that, under the constraint of 10 kcal (mol^−1^ Å^−2^) in a canonical ensemble (NVT), each system was heated from 0 K to 300 K in 300 ps and was further subjected to a 700 ps equilibration run. Finally, a total of 5 μs cMD was performed for each system in both an isothermal-isobaric ensemble (NPT) condition and a periodic boundary condition. During the process of MD simulation, the particle mesh Ewald method was utilized to model the long-range electrostatic interactions, whereas a cutoff of 10 Å was employed to simplify the short-range electrostatic interactions and van der Waals force calculations [49]. In addition, the SHAKE method was conducted to constrain bond interactions involving hydrogen [50].

### 2.4. Principal Component Analysis (PCA)

PCA was carried out to capture the essential motions and characterize overall conformational transitions within each system. In PCA, the covariance matrix of Cα atoms was diagonalized to generate a new set of eigenvectors (also called PC), which described the system motions. The eigenvalue of each PC was related to the mean square fluctuation throughout the system’s trajectory projected along with that PC, therefore the first sorted PC (PC1) corresponded to the most dominant amplitude motion within the system, and the dynamics of the system projected along PC1 was referred as “essential dynamics” [51]. In this work, each system had its trajectory stripped down to only the Cα atoms and superposed onto a reference structure. First, to visualize the major motions within each system using porcupine plots, the sampled conformations within the trajectories were projected into the collective coordinate space defined by PC1, with reference to the starting structure of each system, respectively. Next, to identify differences in the essential structural-dynamic properties of EGFR among different systems, each system had its trajectory superposed onto the same reference structure of the “Src-like” inactive state (PDB ID: 2GS7) and projected along with the first two PCs (PC1 and PC2), so that the motions (with ligands or not) were all projected onto the same set of eigenvectors to achieve comparability.

### 2.5. Community Network Analysis

The dynamic cross correlation matrix (DCCM) was applied to present the correlation between EGFR residues, and the correlation coefficient $C_{ij}$ was calculated through the cMD trajectories based on Equation (1):

(1) $$C_{ij}=\left(\Delta r_{i}\times \Delta r_{j}\right)/{\left(\left\langle \Delta r_{i}^{2}\rangle \times \langle \Delta r_{j}^{2}\right\rangle \right)}^{1/2}$$

In the equation, $\Delta r_{i}$ and $\Delta r_{j}$ represent the atomic displacement vectors for $C_{\alpha }$ atoms *i* and *j*, respectively, while $C_{ij}$ represents the fluctuation correlation between two residues. The correlation data were further applied to weight edges and calculate the edge distance $D_{ij}$ by Equation (2), which indicates the possibility of information flow:

(2) $$D_{ij}=-\log\left(\left|C_{ij}\right|\right)$$

The community network was defined as a set of nodes, which derived from the *C* atoms within EGFR, connected by $C_{ij}$ weighted edges between two nodes that stay adjacent within a cutoff distance of 4.5 Å for at least 75% of the trajectory. The shortest paths were calculated utilizing the Floyd-Warshall algorithm, and the number of pairwise shortest paths cross a given edge was identified as the edge betweenness [52]. A community was defined as a set of nodes that densely interconnect with each other. Based on the betweenness information, the distribution of communities was decided and optimized using the Girvan-Newman algorithm [53]. Communities containing residues less than three were discarded. The optimal paths between node pairs were calculated to reflect inter-community communication, during which all edges connecting the communities were identified and those with the highest betweenness were selected.

### 2.6. Energy Coupling Score Calculation

The molecular mechanisms generalized Born surface area (MM-GBSA) energy decomposition scheme was performed on the corresponding cavities on EGFR^L858R/T790M^ in both the osimertinib-loaded (*apo*) and the osimertinib-unloaded (*holo*) systems throughout their trajectories. To compare the residue-residue interactions for one cavity in the two systems, the energy decompositions within a pocket were calculated for residue pairs separated by at least three amino acids in the sequence based on Equation (3):

(3) $$E=E_{int}+E_{eel}+E_{vdw}+G_{pol}+G_{sas}$$

In the equation, $E_{int}$ represents internal energy, $E_{eel}$ represents electrostatic energy, $E_{vdw}$ represents van der Waals energy, $G_{pol}$ is the polar solvation free energy, and $G_{sas}$ is the solvent accessible surface energy.

The energy decomposition values were calculated and averaged over 5000 snapshots captured from the MD simulation trajectories of *apo* and *holo* systems, respectively.

## 3. Results

### 3.1. Orthosteric Osimertinib Binding Induced a Conformational Transition of EGFR^L858R/T790M^ by Departing the “Src-like” Inactive State

To reveal the detailed mechanism of orthosteric inhibitor binding that enhanced the potency of allosteric ligands in the EGFR mutant, each 5 μs large-scale MD simulation was performed on three systems, including the *apo* EGFR^L858R/T790M^, EGFR^L858R/T790M^ in complex with osimertinib (EGFR^L858R/T790M^–osimertinib), and EGFR^L858R/T790M^ complexed with both osimertinib and JBJ (EGFR^L858R/T790M^–osimertinib–JBJ). Experimental results revealed that the enhancement of allosteric binding was independent of the dimerization level of EGFR in cells. Therefore, it was assumed that osimertinib improved JBJ affinity via conformational regulation in EGFR monomers, instead of inhibiting dimerization to raise the proportion of αC-out conformers [20]. Accordingly, the starting structure of EGFR^L858R/T790M^ in the two systems EGFR^L858R/T790M^ and EGFR^L858R/T790M^–osimertinib stemmed from the crystal structure of EGFR monomeric form (PDB ID: 4I1Z), which was characterized as αC-out (the K745-E762 salt bridge was broken, while the E762-K860 salt bridge was not formed yet), A loop-open (the conserved residue Y891 positioned the backbone of the phosphorylation site Y869 via salt bridges), and DFG-out (Figure S1) [46]. The crystal structure of EGFR^L858R/T790M^ monomer adopted a locally disordered state (the αC helix is placed out, whereas the A loop remains in an active-like open state), instead of a quintessential active state normally found in oncogenic mutants [54]. By contrast, the starting structure of the EGFR^L858R/T790M^–osimertinib–JBJ system came from a JBJ-loaded EGFR^WT^ crystal structure, which presented an “Src-like” inactive state induced by the allosteric inhibitor (Figure S1) [20,55].

For every snapshot of the trajectories, the root-mean-square deviations (RMSD) of all Cα atoms were calculated, referring to the starting structure of each system (Figure S2). The RMSD analysis revealed that all three systems reached equilibrium in 100 ns. For the conformers in equilibrium (100–5000 ns), the RMSD values of EGFR^L858R/T790M^, EGFR^L858R/T790M^–osimertinib, and EGFR^L858R/T790M^–osimertinib–JBJ systems were 2.88 ± 0.51 Å, 2.75 ± 0.24 Å, and 2.98 ± 0.44 Å, respectively. The rather close RMSD values of different systems indicate minor overall conformational alterations of EGFR^L858R/T790M^ in each system during simulations, which implies a relatively stable overall structure of the kinase domain. Therefore, instead of causing major conformational changes, the orthosteric and allosteric ligands stabilized the overall structure of EGFR, leading to the reduction in the standard deviation of RMSD (from 0.51Å to 0.24 Å and 0.44 Å, respectively).

Next, the root-mean-square fluctuations (RMSF) were calculated to reveal the mobility of different regions (Figure S3A). Major fluctuating functional regions that differed across various systems included the P loop, the β3-αC loop, the αC helix, the αC-β4 loop, and the A loop (Figure S3A). The per-residue RMSF difference between EGFR with and without osimertinib was further calculated and projected onto the initial EGFR structure for intuitive visualization (Figure S3B). Upon binding of osimertinib, the RMSF values of the above-mentioned crucial regions exhibited considerable changes that the P loop, the β3-αC loop, the αC-β4 loop, and the A loop were significantly stabilized, while the αC helix became more fluctuated instead. The RMSF differences at various functional regions topologically distal from the orthosteric site suggested that in addition to directly competing with ATP binding, osimertinib also triggered functional alterations within EGFR by modulating the overall structures including allosteric regions. Further inhibition with JBJ led to general stabilization at all these regions, denoting that coadministration with allosteric and orthosteric inhibitors greatly stabilized the functional regions of EGFR^L858R/T790M^ in their inactive states, in agreement with the experimental results of an enhanced efficacy (Figure S3A).

In order to characterize and compare the dominant conformations among different complexes, principal component analysis (PCA) was performed for each system to determine the global conformational transition patterns of EGFR, which was plotted using the two most representative collective principal components (PC1 and PC2). Since the two principal components are calculated without regard to biological significance, they present more objective information to picture the comprehensive structural shifts in the system. Distinct conformations were detected in EGFR with or without the presence of osimertinib. Porcupine plots were first constructed to graphically visualize the dominant movements of different regions in EGFR. The protein dynamics were projected along with PC1 onto the starting structure of each system for intuitionistic description of the major subdomain movements during simulations (Figure S4). The principal dynamic motion of the EGFR^L858R/T790M^ system mainly concentrated on the β3-αC loop, the N-terminus of αC helix, and the middle part of the A loop, but the αC helix remained an αC-out conformation, consistent with the fluctuation analysis results. To further describe the essential eigenvector, the β3-αC loop moved forward in adjacence with the lifted A loop, the N-terminus of αC helix underwent a disordering process, and the A loop moved towards the N-lobe. Accordingly, we assumed a trend towards the “Src-like” inactive state in the *apo* system. In the EGFR^L858R/T790M^–osimertinib system, the full-length αC helix moved inward and the C-terminus of A loop, which was close to the orthosteric site, extended outward to accommodate the binding of osimertinib, but the A loop fixed in the open conformation, very similar to the active state of EGFR. In the EGFR^L858R/T790M^–osimertinib–JBJ system, the overall structure was notably stabilized by the allosteric inhibitor, especially the αC helix which was locked in an out state, while the closed A loop remained its flexible nature.

**Figure 2:**
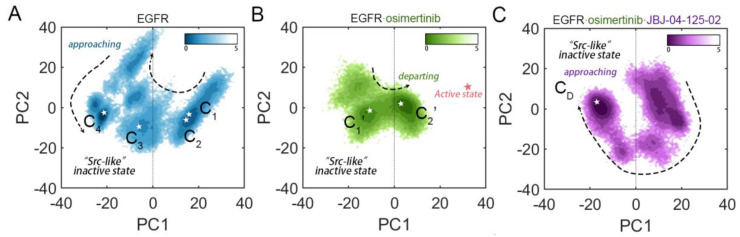
PCA analysis of EGFR^L858R/T790M^ catalytic domain and projection of representative structures. Projection of trajectories along with the first two collective principal components (PC1 and PC2) of EGFR in the (**A**) EGFR^L858R/T790M^, (**B**) EGFR^L858R/T790M^–osimertinib, and (**C**) EGFR^L858R/T790M^–osimertinib–JBJ-04-125-02 systems. The panel is divided into regions of “approaching” (PC1 value shifts from positive to negative) and “departing” (PC1 value shifts from negative to positive) the “Src-like” inactive state along the PC1 axis. Projection of representative structures, including clusters and the active state, is shown in stars. The unit of free-energy values is kcal/mol.

To compare the essential dynamic properties of EGFR among different systems, each system had its trajectory superposed onto the same reference structure of “Src-like” inactive state (PDB ID: 2GS7) [55]. The free energy surface (FES) was then projected along PC1 and PC2 onto a two-dimensional space to visualize the conformational dynamics of EGFR (Figure 2). Therefore, the major dynamics projected along PC1 can reveal the approaching (PC1 value shifts from positive to negative) or departing (PC1 value shifts from negative to positive) motions relative to the αC-out and the A loop closed “Src-like” inactive state. As validated by the control system of EGFR^L858R/T790M^–osimertinib–JBJ, in which the PC1 value shifted from positive to negative, we identified distinct global motions in different systems, with the *apo* EGFR^L858R/T790M^ approaching the “Src-like” inactive state, while the osimertinib-loaded EGFR^L858R/T790M^ departing.

It was previously reported that double-mutant EGFR^L858R/T790M^ retained some characteristics of the single mutations L858R and T790M, however, it showed many other features due to the synergistic effect of the combined mutations. Four relatively stable conformations (1 active state and 3 inactive states) were discovered in the *apo* EGFR^L858R/T790M^, and in particular, the energy barrier for the “open-to-closed” transition of the A loop was drastically lowered, which caused greater flexibility populating active and inactive states [7]. Since the EGFR^L858R/T790M^ with or without osimertinib binding shared the same starting structure of the locally disordered state, although the complete inactive-to-active transition of EGFR^L858R/T790M^ was not observed, the opposite conformational transition pathways adopted by the two systems suggested altered energy barriers among various conformations, and thus led to a renewed dynamic ensemble. Collectively, the introduction of orthosteric inhibitor osimertinib generated an altered dominating conformation of EGFR^L858R/T790M^ by departing the “Src-like” inactive state. The inward movement of the αC helix observed in the EGFR^L858R/T790M^–osimertinib complex would exert dramatic structural changes at the allosteric MEK-pocket, consistent with the experimental evidence of an enhanced JBJ affinity in response to osimertinib binding.

### 3.2. Osimertinib Binding Stabilized an Intermediate State Characterized by a Unique αC-in, A Loop Open, and DFG-out Conformation

Considering that the transition of departing the “Src-like” inactive state of EGFR^L858R/T790M^ induced by osimertinib, we next explored more detailed conformational characteristics over functional regions. The universally conserved residue K745 coordinates the α- and β-phosphates of ATP and anchors the αC helix via a salt bridge with E762, thereby playing a pivotal role in the activation of EGFR. On the other hand, the A loop, starting with the conserved DFG motif and ending at the αF helix, contains the phosphorylation site Y869 that is the centerpiece of protein allosteric regulation. It undergoes an order-to-disorder transition during the inactivation process of kinases.

Protein kinases including EGFR contain hydrophobic regulatory and catalytic spines required for the assembly of active enzymes. To verify if the major cluster C_2′_ in the osimertinib-loaded system represents an active state, we further analyzed the DFG motif and the regulatory spine (R spine). The R spine, consisting of four hydrophobic residues (H835, F856, M766, and L777) that span both the N and C lobes of EGFR, is formed by hydrophobic contacts. In the inactive state, the R hspine features a kink or discontinuity which leads to loss of catalysis function. While the conversion from inactive to active EGFR is accompanied by the assembly of the R spine. In the T790M mutant, the hydrophobic residue M790 participates in the assembly of the R spine, validated as an aberrant activation mechanism in oncogenesis. Identical with the starting structure, the dominant structure of *apo* EGFR^L858R/T790M^ and EGFR^L858R/T790M^–osimertinib systems both adopted the DFG-out conformation, resulting in a broken R spine (Figure 4A,B). In the EGFR^L858R/T790M^–osimertinib–JBJ system, despite the inward rotation of D858, the R spine was also broken by the allosteric ligand and the repositioned M766 (Figure 4C). Since the R spine was completed and assembled in none of the systems, they were all identified as catalytically inactive. However, C_2′_, induced by osimertinib disturbance at the orthosteric site, was characterized as αC-in, A loop open, and DFG-out, highly resembling the structure of an Abl intermediate state recently reported by Xie et al. [57]. They performed nuclear magnetic resonance spectroscopy to speculate the transition pathway of Abl activation and discovered a novel inactive conformation (I1), characterized as αC-in, A loop open, and DFG-out. Herein, we proposed that such intermediate conformation existed in the *apo* EGFR as well, and osimertinib stabilized this unique state by rendering the β3 strands in the N-lobe to approach the αC helix, thus facilitating the formation of the K745-E762 salt bridge. Moreover, the inward movement of the αC helix resulted in M766 rotation, which filled into the space between L777 and F856, contributing to the assembly of the R spine. We calculated the sum of the M766-M790 and M766-H835 distance, and discovered a significant decrease from 16.49 Å to 14.77 Å in the sum upon osimertinib loading (Figure 4D). The declined distance reflected a more assembled state of the R spine, which would engender stronger hydrophobic attraction to promote the proper orientation of F856, facilitating complete assembly of the R spine. Additionally, we projected the active structure (PDB ID: 2GS2) [53] along PC1 and PC2 of the EGFR^L858R/T790M^–osimertinib system, and C_2′_ was found located along the activating pathway, illustrating our hypothesis of C_2′_ as a unique intermediate conformation (Figure 2B).

To validate our simulation results and identify detailed structural differences between the *apo* EGFR^L858R/T790M^ and EGFR^L858R/T790M^–osimertinib systems, we compared the representative structures of these two systems with the crystal structure extracted from Protein Data Bank (Figure 5), respectively. For the unavailability of EGFR^L858R/T790M^–osimertinib structures, we selected EGFR^L858R/T790M/C797S^–osimertinib (PDB ID: 6LUD) as a reference, which was reported to show similar conformational effects with only lower affinity regarding EGFR^L858R/T790M^ [58]. The crystal structure of EGFR^L858R/T790M/C797S^–osimertinib adopted an active conformation as we discussed above, with an extended A loop and a closely interacting ion pair K745-E762. In addition, other crystal structures of EGFR complexed with osimertinib, including EGFR^WT^ (PDB ID: 4ZAU) [59], EGFR^L858R^ (PDB ID: 6JWL) [56], and EGFR^T790M^ (PDB ID: 6JX4) [56] all exhibited an active conformation, strengthening the reliability of our simulation results [56,59]. Upon alignment of EGFR^L858R/T790M^–osimertinib with EGFR^L858R/T790M/C797S^–osimertinib, the low RMSD value (0.94 Å) of EGFR reflects no significant difference between the overall structures of these two complexes. With detailed structural analysis zooming into several important regions, some subtle distinctions emerged. In the C_2′_ conformation, F723, the bulkiest residue located in the middle of the P loop, swung outward by ~12 Å toward the αC helix, while it was flipped and positioned right at the top edge of the orthosteric site in the crystal structure (Figure 5A). The translocated F723 not only acted as a wedge to push the N-terminus of αC helix away from adopting its αC-in conformation, but also posed a massive steric hindrance against the inward rotation of F856, resulting in the kinked αC helix and DFG-out state. Furthermore, the outward location of F856 observed in EGFR^L858R/T790M/C797S^–osimertinib structure agreed with the experimental results that the affinity of JBJ was enhanced by osimertinib, since type IV inhibitors including JBJ require a folded aromatic residue to create a cavity between the P loop and the αC helix [60]. Despite the differences found in the DFG motif, the A loop conformations were highly consistent across the two structures. Importantly, the key phosphorylation site Y869 was exposed on the protein surface, stabilized by the conserved salt bridges with Y891 and other electrostatic interactions with K860 and R836, thus contributing to the phosphorylation at Y869. In a previous study on another kinase Akt, it was revealed that the binding of TKIs to the orthosteric site was sufficient to cause hyperphosphorylation of Akt in the absence of any pathway feedback effects [61]. Herein, our simulation results revealed a possible explanation that the TKIs targeting Akt, similar to osimertinib with EGFR, stabilized the open state of the A loop, thus positioning the key residue in its proper place for phosphorylation.

**Figure 3:**
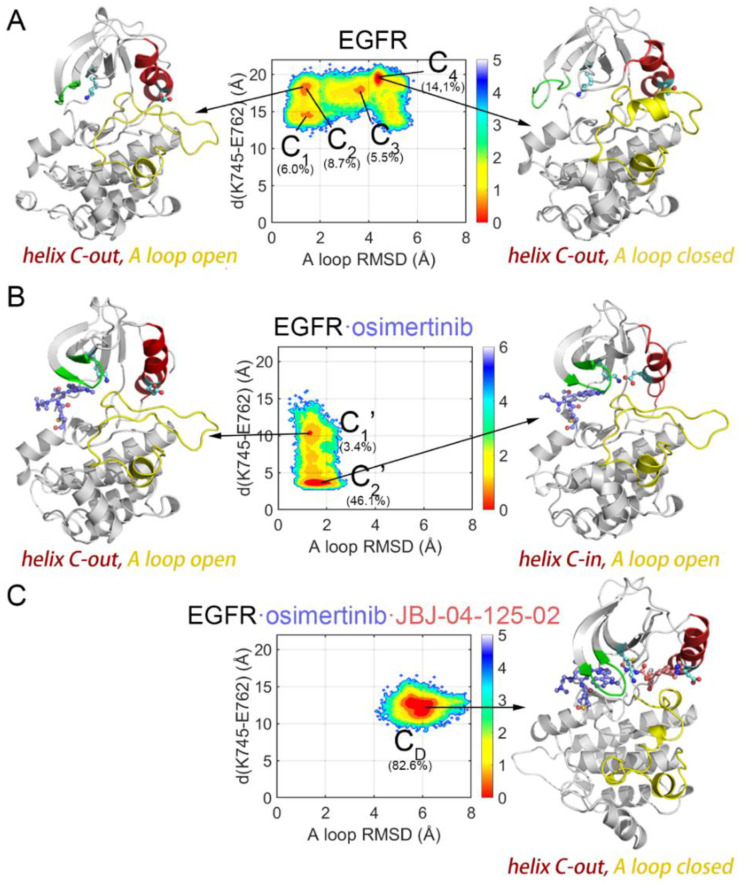
Conformational free energy surface of EGFR^L858R/T790M^ relevant to activation/inactivation. The landscape is generated using the distance between K745 (zeta nitrogen) and E762 (delta carbon), and the RMSD in the A loop in the (**A**) EGFR^L858R/T790M^, (**B**) EGFR^L858R/T790M^–osimertinib, and (**C**) EGFR^L858R/T790M^–osimertinib–JBJ-04-125-02 systems. The dominant conformational rearrangements in the representative structures of the free energy minima are shown with the αC helix colored in red, the K745-E762 residue pair in blue, the A loop in yellow, and the P loop in green. The unit of free-energy values is kcal/mol.

**Figure 4:**
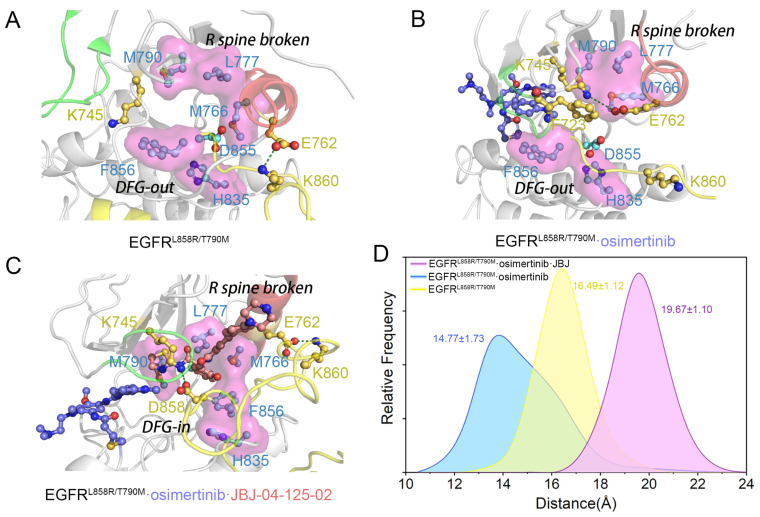
Structural analysis of the DFG motif and the R spine. Atomistic details of the DFG motif and the R spine in the (**A**) EGFR^L858R/T790M^, (**B**) EGFR^L858R/T790M^–osimertinib, and (**C**) EGFR^L858R/T790M^–osimertinib–JBJ-04-125-02 systems. (**D**) Distance sum of M766-M790 and M766-H835 calculated from conformations sampled during the MD simulations of EGFR^L858R/T790M^ (yellow), EGFR^L858R/T790M^–osimertinib (blue), and EGFR^L858R/T790M^–osimertinib–JBJ-04-125-02 (purple).

Two collective variables (CVs), CV1 and CV2, were chosen based on the formation of the K745-E762 salt bridge and the conformation of the A loop. CV1 was defined as the distance between the coupled residues, which characterized the displacement and rotation of the αC helix shifting between αC-in and αC-out conformations. While CV2 was defined as RMSD of the A loop relative to the open conformation, exhibiting its structural alterations towards the disordered state. The FES was then projected along CV1 and CV2 to compare the conformational landscapes of EGFR in each system (Figure 3). The *apo* EGFR^L858R/T790M^ system exhibited a relatively wide-spread distribution of conformational ensembles on the FES, with four dominant conformations highlighted (Figure 3A and Figure S5). Conformational ensembles of EGFR^L858R/T790M^ were further clustered into 4 states (C1, C2, C3, and C4) in a stepwise manner C1→C2→C3→C4. The change from C1 to C2 can be attributed to the outward rotation of K745, which derived from the broken K745-E762 salt bridge and the subsequent loss of the anchoring function in K745, leading to an increased distance (from ~14 Å to ~18 Å) between the already separate residues. Furthermore, the rotated K745 partially occupied the ATP-binding site and induced a kinked P loop, both impeding ATP binding. The following dynamic steps from C2 to C4 were characterized by the rise in A loop RMSD (from ~1.5 Å to ~4.4 Å), indicating a marked conformational transition, which was already observed in RMSF and PCA analysis (Figure 2A, Figure 3A, and Figure S5). The cluster structure revealed that the A loop underwent an order-to-disorder process and a short helix was formed in the middle of the A loop (^167^KEYHHAEG^173^), thus presenting an αC-out and A loop closed conformation. Although, to our knowledge, this A loop conformation was not captured in crystal structures of EGFR, similar conformations have been previously reported in other MD simulation studies as an intermediate state observed in the transition process to the “Src-like” inactive state [56]. Albeit the identical “Src-like” inactive state had not been achieved in the trajectory, the motions of the αC helix outward rotation and the A loop disordering implied that the starting structure of EGFR^L858R/T790M^ without osimertinib favored the classical autoinhibitory state, showing an easier transition towards catalytic inactivation instead of activation. Additionally, all the cluster structures were projected onto the FES in PCA, confirming a stepwise transition of EGFR approaching the “Src-like” inactive state (Figure 2A). In contrast, in the osimertinib-loaded system, dramatic change in the FES was witnessed. The surface appeared to be less extended, and two main conformations were discovered (C_1′_ and C_2′_), with RMSD values of approximately 1.3 Å and 1.7 Å in the A loop and K745-E762 distances of approximately 10.3 Å and 3.6 Å (Figure 3B). The transition from C_1′_ and C_2′_ can be attributed to the inward rotation of K745 induced by its interaction with the nearby osimertinib, followed by the formation of the K745-E762 salt bridge and the inward movement of the αC helix. Meanwhile, unlike the *apo* system, the A loop remained in an open state. The two clusters were also projected along PC1 and PC2, validating that osimertinib induced an altered FES and lowered the energy barriers departing the “Src-like” inactive state. Furthermore, additional binding of JBJ led to a highly dominating “Src-like” inactive state (C_D_, with RMSD value of ~5.9 Å and distance of ~12.2 Å), which was closer to the *apo* system instead of the osimertinib-loaded system (Figure 3C). Despite the inward K745 caused by osimertinib loading, JBJ directly sabotaged the interaction between K745 and E762, locking the αC-out state. Moreover, the allosteric inhibitor was validated to form salt bridges with F856 and E865, leading to the stabilization of a disordered A loop [20].

**Figure 5:**
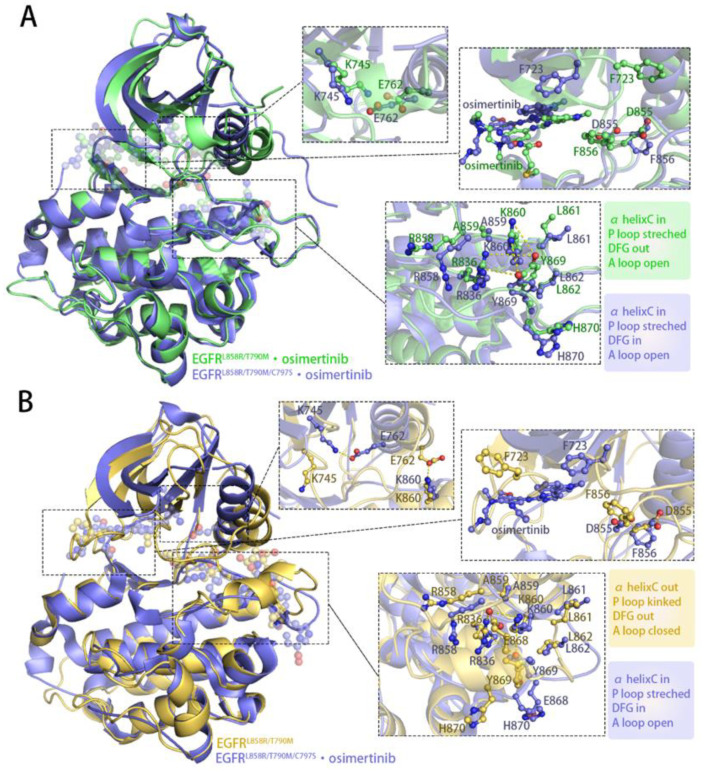
Structure of EGFR mutants in complex with osimertinib. (**A**) The representative structure of the EGFR^L858R/T790M^–osimertinib system (lime) superimposed on the crystal structure of EGFR^L858R/T790M/C797S^–osimertinib (slate, PDB ID: 6LUD). The magnified views highlight the similarities (e.g., αC helix and A loop) and differences (e.g., DFG motif) in the disposition of key structural elements between the two structures. (**B**) The representative structure of the EGFR^L858R/T790M^ system (yellow) superimposed on the crystal structure of EGFR^L858R/T790M/C797S^–osimertinib (slate). The magnified views highlight the differences in the disposition of key structural elements between the two structures.

We next compared the EGFR^L858R/T790M/C797S^–osimertinib structure with the C_4_ cluster we obtained from EGFR^L858R/T790M^ simulations (Figure 5B), in which case, more prominent distinctions were unraveled with an RMSD value of 1.98 Å. Aside from a broken K745-E762 salt bridge and a newly formed E762-K860 salt bridge, F723 at the P loop shifted outward by ~10.8 Å, leaving a much larger space for F856 rotation. This major shift in the P loop is induced by the steric exclusion of the large osimertinib. The addition of osimertinib occupies the ATP-binding site and directly pushes the β strands towards the αC helix, while in the *apo* complex, the side chain of F723 is free to extend into the orthosteric site, thus presenting a large shift. However, F856 flipped into the catalytic site to occupy the ATP-binding site. Meanwhile, D855 oriented in the opposite direction unsuitable to bind the implicated magnesium ion, also obstructing the binding of ATP. On the other hand, Y869 folded into a short helix and rotated approximately 180° away from the protein surface. The conserved salt bridges between Y869 and Y891 were broken, while the E868-K860 and E868-R836 salt bridges were formed to stabilized the helix for substitution (Figure 5B). Therefore, C_4_ adopted a fully inactive conformation with the four key elements all being in states not compatible with either phosphorylation or ATP loading.

The activation of EGFR is accompanied by electrostatic exchanges involving the breaking and formation of distinct sets of conserved hydrogen bonds across the kinase domain, including K745-E762, E762-K860, R836-Y869, and D837-N842. Herein, we analyzed the allosteric dynamics of these key salt bridges upon osimertinib binding. Compared with the structure of EGFR^L858R/T790M^ cluster, the representative structure of the EGFR^L858R/T790M^–osimertinib system features the broken E762-K860 salt bridge and the stable K745-E762 salt bridge instead. This shift of hydrogen bond explains the αC-in conformation at the allosteric site. Moreover, the essential R836-Y869 salt bridge, which connects the HRD motif of the catalytic loop and the activation segment, was formed upon osimertinib loading, and the salt bridge between R836 and E868 was consequently broken. Such a shift implies that osimertinib induces the explosion of Y869 for phosphorylation at the activation loop, which is required for kinase activity. Additionally, a network of hydrogen bonds was formed in the EGFR^L858R/T790M/C797S^–osimertinib structure, encompassing D855 of the DFG motif, D837 of the HRD motif, and N842 of the catalytic loop. The catalytic base D837 orients the tyrosyl group of the substrate protein in a catalytically competent state and functions as a base to abstract a proton from the tyrosine residue, thereby facilitating its nucleophilic attack of the γ-phosphorus atom in MgATP. The salt bridge network induced by osimertinib was stabilized by the DFG-out conformation and further improved the catalytic activity of EGFR.

Taken together, osimertinib binding stabled a potential intermediate state with a unique αC-in, A loop open, and DFG-out conformation, thus shifting the transition direction towards the activation state. Namely, the perturbation of orthosteric ligands not only posed effects on nearby regions, but also spread its influence into topologically distant areas, thus exerting an impact on the overall functions of the target protein. As such, it is feasible to precisely regulate the allosteric sites both structurally and functionally with orthosteric ligands, providing theoretical support for the dual-targeting therapeutic strategy.

### 3.3. Stepwise Dynamics of EGFR Transition towards the “Src-like” Inactivation State

**Figure 6:**
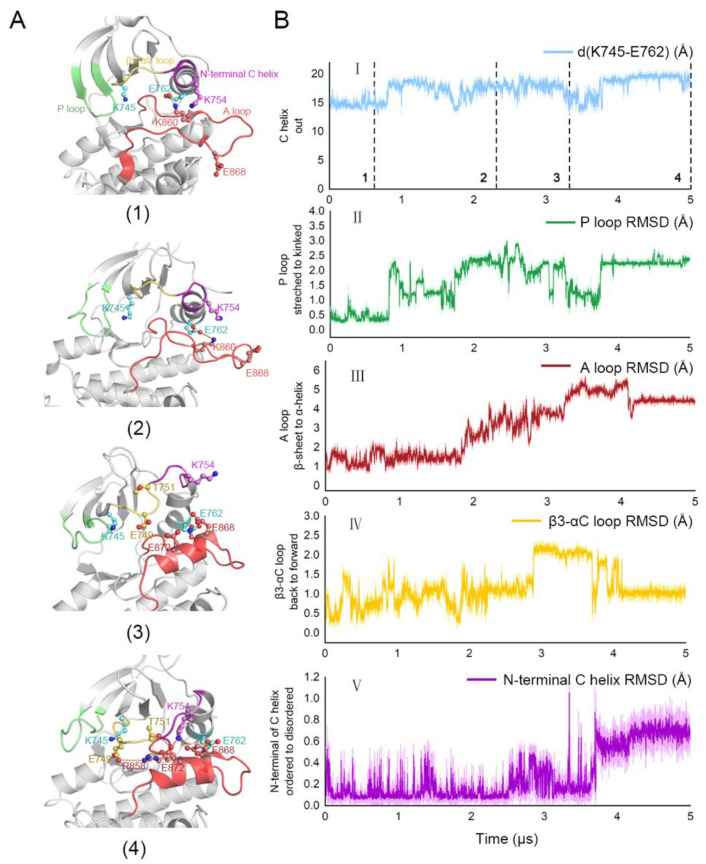
A concerted process of EGFR transition towards the “Src-like” inactivation state. (**A**) Four structures are extracted from the trajectory to depict the stepwise transition of EGFR from the locally disordered state in (1) to the “Src-like” inactive state in (4). (**B**) Kinetics of several molecular switches as inferred from the 5 μs MD trajectory. The trace exhibit variation of five key parameters. (I) The salt bridge distance between K745 and E762, indicating an αC-out conformation throughout the trajectory. The black dashed lines correspond to the structures plotted in (**A**). (II) RMSD of the P loop, indicating a stretched to kinked transformation. (III) RMSD of the A loop, indicating an order-to-disorder transition. (IV) RMSD of the β3-αC loop, indicating a forward swinging. (V) RMSD of the N-terminus of αC helix, indicating an order-to-disorder transition. All RMSD values were calculated using heavy atoms and were in reference to the starting structure of EGFR^L858R/T790M^. Colors match the structural features in (**A**), while darker colors indicate moving averages across 10 frames and lighter colors indicate the corresponding standard deviations.

To unravel the conformational transition pathway from the locally disordered state to the “Src-like” inactive state within EGFR^L858R/T790M^, we analyzed the dynamic process based on several structural parameters (the distance between K745 and E762, RMSD of the P loop, A loop, β3-αC loop, and N-terminal αC helix) throughout the trajectory of the *apo* EGFR^L858R/T790M^ system. Within our simulations, the EGFR^L858R/T790M^ system underwent three major stages, including a stable stage with minor structural alterations (approximately 0~800 ns), a remarkably dynamic and constantly evolving stage (approximately 800~4000 ns) when the overall conformation approached the “Src-like” inactive state, and another considerably stabilized stage (approximately 4000~5000 ns). Herein, we identified a stepwise manner of EGFR transforming (Figure 6). At the beginning of the second stage (800 ns), a sudden but sharp increase can be speculated almost simultaneously in K745-E762 distance and P loop RMSD, illustrating that the motion of P loop and αC helix was highly coupled (Figure 6A(1,2),B). We assumed that this correlation was induced by the outward rotation of E762, which destabilized the β strands via a mitigated anchor function, hence K745 rotated towards the orthosteric site and disturbed the stretched P loop. Accordingly, the structural crosstalk between the allosteric site (αC helix) and the orthosteric site (P loop) was realized. Next, during the transforming stage, a conspicuous change in the A loop occurred at ~2000 ns, followed by another noteworthy motion in β3-αC loop at ~3000 ns (Figure 6A(3,4)). Since the newly formed helix was first stabilized by residues in the αC helix and the N-terminus of A loop (e.g., E868-K860, E868-R836, and E872-R858 salt bridges), we proposed that the short helix was likely to be initiated by the outward movement of αC helix and a subsequent disturbance at the local structure (e.g., K860 rotation) in the A loop. The posterior forward swinging of the β3-αC loop could be further attributed to the approaching of certain amino acids on the A loop. Accordingly, the translocation of β3-αC loop induced a complicated electrostatic interaction network among multiple ion pairs, which further stabilized the short helix. Finally, the N-terminus of αC helix underwent a disordering process (~3800 ns), during which the helix broke down into a loop, and directly promoted the system to enter a stable stage (Figure 6A(4)). With an expanded interface between N-lobe and C-lobe, the newly generated loop formed key salt bridges with the A loop, including K754-E868 and K754-E872, which contributed to the stabilization of the short helix. Moreover, the prolonged linkage loop between the β3 strand and the αC helix allowed a greater distance between the two regions within N-lobe, in consistence with the relatively long distance between the allosteric and orthosteric sites observed in the “Src-like” inactive state. In general, we explored the stepwise dynamics during the conformational transition of EGFR^L858R/T790M^, which implied a concerted allosteric signaling pathway and gave us a clue about the structural correlation among the orthosteric site, the JBJ binding site, and the A loop.

### 3.4. Reversed Allosteric Signaling Pathways within EGFR^L858R/T790M^ Catalytic Domain

**Figure 7:**
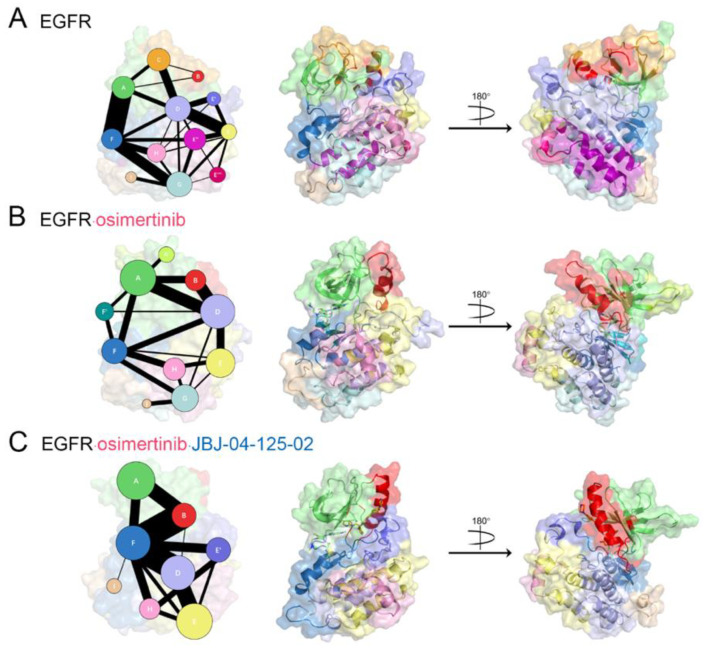
Community network representations. The maps of community network in the (**A**) EGFR^L858R/T790M^, (**B**) EGFR^L858R/T790M^–osimertinib, and (**C**) EGFR^L858R/T790M^–osimertinib–JBJ-04-125-02 systems are depicted. Each sphere represents a corresponding community whose number of residue components is indicated with the sphere area. While the sticks connecting different spheres visualize the inter-community connections, and the thickness of these sticks is proportional to the value of edge connectivity.

We next conducted to explore the propagation pathway of allosteric signals, utilizing a community analysis based on the Girvan–Newman algorithm, which provided a quantitative estimate of the allosteric coupling among communities [53]. Residues within a cutoff distance of 4.5 Å for at least 75% snapshots throughout the trajectory were distributed into the same community, which were deemed as a synergistic functional unit during the multiple microsecond simulations of EGFR^L858R/T790M^. While the intercommunity information flow was defined as the time-averaged connectivity between communities, clearly delineating the pathway and strength of allosteric crosstalk. Furthermore, the visualized community network enabled an intuitive comparison of EGFR^L858R/T790M^ signaling among distinct systems (Figure 7).

From a general view, the global complexity of community connection decreased, and the number of communities dropped upon ligand binding, with 13 closely interacted communities detected in the *apo* system, 10 in the EGFR^L858R/T790M^–osimertinib system, and only 8 in the EGFR^L858R/T790M^–osimertinib–JBJ system, denoting a topological solidification function of both the orthosteric and allosteric inhibitors. Namely, the binding of inhibitors considerably reshaped the topology of several communities that they were recategorized and merged into the same community. This implied that some less important connections were quenched upon inhibitor binding, but specific signaling pathways were promoted to transmit effects of the ligands.

In the *apo* EGFR^L858R/T790M^ system, Community A was composed of the majority of β strands located close to the C-lobe, including the P loop, while the rest was split from Community A, and together with the N-terminus of αC helix, shaped Community C (Figure 7A). The αC helix was separated into three different communities, with the major part in the middle forming Community B. The direct information flow between Community A and B was rather weak, while the function of Community C was to facilitate the desirable position of the γ-phosphate of ATP and the αC helix, which served as a communication link bridging Community A and B. Community D that was situated at the interface between N-lobe and C-lobe consisted of important segments from both lobes, encompassing the C-terminus of αC helix, the αC-α4 loop, half of the A loop (855DFGRAKLL862), and the C-terminus of αE helix. This community was localized at the center of EGFR and formed strong connections with all large communities, in line with its identified function as a core regulatory “hub” [62]. Next, four segregated communities, E, E′, E″, and E‴, were joined to constitute the activation segment of EGFR, among which Community E played a central role as a signal integration motif to transmit information within the segment. Community E included a part of the activation segment (^881^MALESILHRIYT^892^), and two crucial residues E884 and T892 form conserved salt bridges with Y869 and R958, respectively, which explained its communication with community E′ and E‴. Community E′ was characterized as the short helix formed in the middle of the A loop, which mainly communicated with Community D and E, consistent with our previous findings in the order-to-disorder transition of the A loop. In company with Community A, Community F also contributed to the construction of the orthosteric binding site. Community F mainly contained the αD helix and β6–β8 strands, which was responsible for properly locating the ATP molecule, especially the γ-phosphate and two magnesium ions, the phosphate is properly positioned for transfer towards the substrate. Moreover, the C-lobe also embodied several minor communities (Community G, H, and I), which have proved associated with substrate binding.

In the EGFR^L858R/T790M^–osimertinib system, a distinct alteration was observed in both topologic features and intercommunity communications throughout EGFR (Figure 7B). We first focused on the orthosteric site, where the binding of osimertinib induced the split of Community F into F (αD helix) and F′ (β6–β8 strands), accompanied by its significantly attenuated crosstalk with Community A. This indicated that the interlobular information flow via the pathway between Community A and F was blocked by the orthosteric inhibitor, counting against the synergistic accommodation for ATP binding. By contrast, the interaction between Community A and D was greatly strengthened, implying an enhanced interlobular signaling path, which may provide a basis for the reversed allosteric effect of orthosteric inhibitors at more distant regions. To further identify the effect of osimertinib binding on the allosteric site, we next zoomed into the N-lobe. Community C explored in the EGFR^L858R/T790M^ disappeared and the related segment was redistributed into Community A and B, leading to the enlargement of these two communities, which contain β1~β5 strands and the integral αC helix, respectively. Such variation in community categorization was consistent with our structural understanding that osimertinib pushed back β1~β3 strands and reduced their distance with β4 and β5 strands. Moreover, according to the inward movement of αC helix, a firmly coiled N-terminus, and its disassociation with the A loop, αC helix gained structural solidity and turned to function as a whole. Despite the lost bridging function by Community C, the direct crosstalk between Community A and B was remarkably improved, which might result from the formation of the K745-E762 salt bridge. Additionally, a markedly strengthened information flow between Community B and Community D emerged upon osimertinib binding. Therefore, in addition to the enhanced crosstalk between separate parts within the N-lobe, a comprehensive reinforcement was also discovered in the interaction between the N-lobe and the junctional area of Community D, validating that orthosteric targeting with osimertinib transported long-distance signals with not only the αC helix, but also the C-lobe. Correspondingly, the dominating functional components in the C-lobe, Community E, E′, E″, E‴ identified in the *apo* system merged into a single Community E that assembled around the activation segment, demonstrating their approaching each other. Nevertheless, the connection between Community D and E was relatively diminished. It was aforementioned that the crosstalk between community D and E′ helped the order-to-disorder transition of the A loop, thus a decline in such connection would lead to a firmly extended conformation of the A loop, which was in agreement with our structural findings. Together, we proposed that orthosteric inhibitors and the ATP-binding site affected the activation segment through an enhanced information flow with the junctional area, which restrained its crosstalk with the A loop and inhibited its order-to-disorder transition.

In the EGFR^L858R/T790M^–osimertinib–JBJ system, we observed increasingly intensive changes in the community crosstalk network (Figure 7C). Since the JBJ-binding site located close to the P loop, the αC helix, and the A loop, the topology of the ambient region was dramatically altered, and resulted in the redistribution of the αC-β4 loop and part of the A loop into Community F. Consequently, Community F replaced Community D as the central regulatory “hub”. Moreover, Community E again separated into E and E′, according to the short helices in the A loop induced by the allosteric inhibitor. The connection among Community A, B, and F was conspicuously enhanced due to the cross-linking function of JBJ. While the information flow between F and E′ was further strengthened in comparison with the *apo* system, supporting our notion that the information transmission from the αC-β4 loop and the N-terminus of A loop to the C-terminus of A loop induced its disordering transformation. On the other hand, the original Community G merged with Community D, which reflected the structural alterations within αE and αI helices. This could lead to the impaired binding of EGFR substrates, further dampening the EGFR activity via dual targeting.

In general, we identified the reversed and forward allosteric signaling pathways among various functional regions, which were enhanced by osimertinib and JBJ, respectively. The characterization of these promoted interactions highlighted Community D (especially the αC-β4 loop and the N-terminus of the A loop) as a core transmission hub. Both orthosteric and allosteric inhibitors regulated the overall conformation by propagating certain signals to distant subdomain areas, during which this interlobular junction region played a central role to connect all major communities.

### 3.5. Identification of Potential Allosteric Pockets Based on Reversed Allosteric Communication

Since the MEK-pocket is the only identified allosteric site in EGFR, revealing other potential allosteric pockets is greatly beneficial for expanding the opportunities to develop novel allosteric inhibitors. Based on the reversed allosteric communication, we demonstrated that distal regions were highly sensitive to orthosteric perturbations. Namely, the binding of orthosteric ligands induced overall dynamics throughout the target protein, leading to possible emergence, stabilization, and conformational alteration at potential allosteric sites, thus providing novel targets for future drug development. Furthermore, since the functional allosteric sites displayed significant correlations with the orthosteric site, it presented a potential strategy to discover a new allosteric pocket depending on its marked coupling with the orthosteric site.

Upon osimertinib binding, two major regions underwent a major conformational transformation, which were regarded as potential allosteric sites, including the cavity between the DFG motif and the αC helix, and the areas surrounding the A loop. The former, also referred to as the “MEK-pocket”, a rather conserved allosteric site across the human kinome, represents the only currently established druggable allosteric pocket in EGFR. In addition, a previous study demonstrated that the occurrence of oncogenic mutations over the kinase domain was highly biased towards specific functional regions, with the highest frequency of oncogenic mutations distributed in the A loop [63]. Such a discovery further validated the A loop as a vital allosteric region, whose mutations can engender malfunction of the whole protein. Therefore, we applied a pocket prediction algorithm focusing on the surrounding areas of the A loop, aiming to detect novel potential allosteric sites for future drug design.

**Figure 8:**
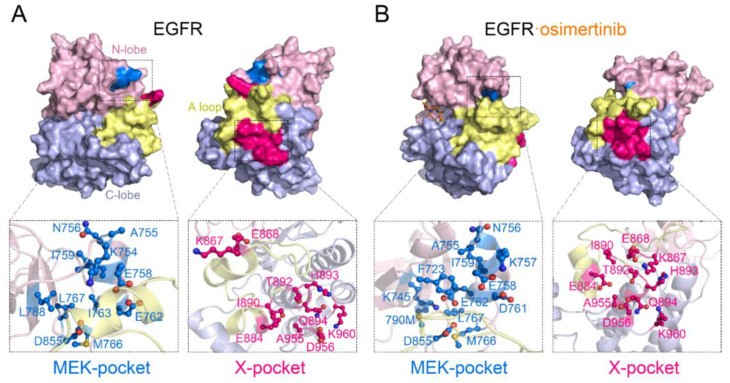
Structure overview of the allosteric pockets. The surface representations of the N-lobe (pink), the C-lobe (lightblue), the A loop (pale yellow), the MEK-pocket (marine), and the X-pocket (hotpink) in the (**A**) EGFR^L858R/T790M^ and (**B**) EGFR^L858R/T790M^–osimertinib systems. The magnified views depicted the critical residues within the allosteric pockets.

First, we utilized D3Pockets, a web server designed for protein pocket analysis, to quantify the stability of the potential binding pockets [64]. As shown in Figure S6A, the grid points that composed the pockets were colored by observed frequencies throughout the trajectory, with the red points representing the most stable regions in a pocket. In the EGFR^L858R/T790M^–osimertinib system, the orthosteric site was stabilized compared with the *apo* system. Surprisingly, the entrance of the MEK-pocket was partially closed due to the inward movement of the αC helix, which indicated that osimertinib may facilitate JBJ binding with a unique “induced-fit” mechanism, instead of direct opening of the MEK-pocket. These observations further evidenced the reversed allosteric communication and the dynamic features of the allosteric site, since it needed to undergo an “induced-fit” process to open the pocket and accommodate allosteric inhibitors. Notably, a novel potential allosteric pocket, named X-pocket, was detected around the A loop and displayed improved stability upon osimertinib binding. X-pocket was positioned between the activation segment and the αE, αF, αH, and αI helices, which appeared to be rather shallow unless it was deepened by an extended A loop. Accordingly, the unique position may enable fine-tuning of the overall EGFR functions via non-ATP nor substrate competitive inhibitors. We further applied Fpocket to retrieve potential binding pockets from the representative structures of the EGFR^L858R/T790M^ and the EGFR^L858R/T790M^–osimertinib system (Figure 8), respectively. The druggability score of the MEK-pocket visibly deteriorated from 0.561 to 0.022 upon osimertinib binding, while the X-pocket exhibited a 230-fold increase from 0.002 to 0.469. Additionally, we noted that the X-pocket in EGFR was roughly analogous to a site previously identified in glycogen synthase kinase 3 (GSK3) that has already been targeted by a number of potent allosteric inhibitors [65,66,67]. Therefore, we assumed the X-pocket as a potential allosteric site and concentrated on it to further characterize the coupling between allosteric and orthosteric sites. The results were compared with the known MEK-pocket for validation (Figure S6B).

**Figure 9:**
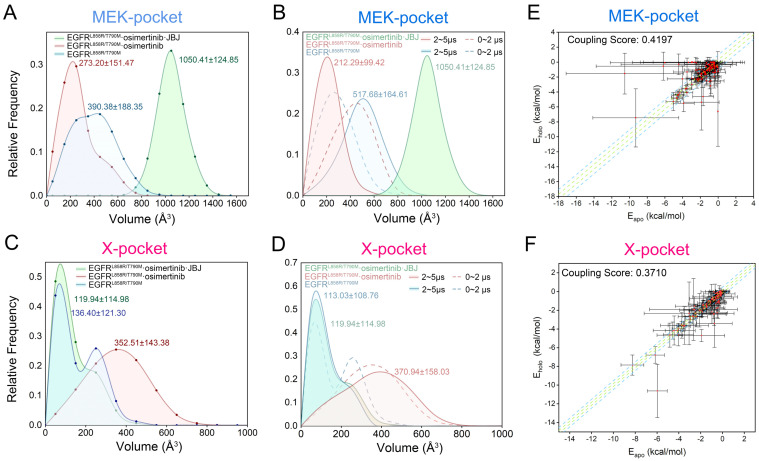
Volume calculations and energy coupling analysis for the allosteric pockets. (**A**) Volume distributions of the MEK-pocket. (**B**) Decomposed volume distributions of the MEK-pocket, with dashed lines and solid lines representing 0~2 μs, and 2~5 μs trajectories, respectively. (**C**) Volume distributions of the X-pocket. (**D**) Decomposed volume distributions of the X-pocket. (**E**) Energy coupling analysis for the MEK-pocket in *apo* and *holo* EGFR^L858R/T790M^. (**F**) Energy coupling analysis for the X-pocket in apo and holo EGFRL858R/T790M. Each dot represents the energy decomposition of a residue pair, while the cross lines represent the corresponding standard deviations. The dots placed in the region between green and blue dashed lines indicate residue pairs with medium energy differences, while the dots placed outside the blue dashed lines indicate residue pairs with large energy differences.

MDpocket was used to track the allosteric pockets and calculate their volume values throughout the MD trajectories [68]. The distribution of MEK-pocket volumes calculated over the entire trajectories in the three systems (Figure 9A) revealed that compared with the fully opened pocket induced by JBJ binding (1050.41 ± 124.85 Å^3^), the MEK-pocket was not completely formed during simulations in neither the *apo* system (390.38 ± 188.35 Å^3^) nor the osimertinib-bounded system (273.20 ± 151.47 Å^3^), indicating the aforementioned “induced-fit” mechanism by allosteric ligands. To better compare the pocket volumes of the dominating structure in each system, we decomposed the more dynamic trajectories of the EGFR^L858R/T790M^ and EGFR^L858R/T790M^–osimertinib systems into two separate parts, containing 0~2 μs and 2~5 μs trajectories, respectively (Figure 9B). The decomposed volume values of MEK-pocket unveiled a notable decrease upon osimertinib binding, with the average volume dropping from 517.68 ± 164.61 Å^3^ to 212.29 ± 99.42 Å^3^, indicating a strong structural correlation between the allosteric MEK-pocket and the orthosteric perturbation. The contrast among the three systems showed clear evidence that the volume of MEK-pocket was highly related to the position of the αC helix, which caused the closure of the pocket by adopting the αC-in state. Considering the experimental results that osimertinib loading improved the binding of allosteric inhibitors to MEK-pocket, it can be estimated that the αC-in state might conversely facilitate the “induced-fit” process at allosteric sites, which also explains the selectivity of JBJ towards the active oncogenic mutants of EGFR. Similarly, volume distinctions and structural changes were discovered in the novel X-pocket (Figure 9C,D). The dominating structures in EGFR^L858R/T790M^ and the EGFR^L858R/T790M^–osimertinib–JBJ systems shared a very similar distribution of X-pocket volume, with an average of 113.03 Å^3^ and 119.94 Å^3^, respectively. While in the EGFR^L858R/T790M^–osimertinib system, the volume sharply increased, surging to an average of 370.94 Å^3^. Therefore, it was illustrated that osimertinib binding stabilized the allosteric X-pocket in a deepened state with the open A loop, thus contributing to ligand targeting.

Since the volume calculation results only deciphered structural coupling between the allosteric and orthosteric sites, we further exploited a recently established quantitative algorithm to delineate the crosstalk in the view of energetics [28]. The calculation model arose from the reversed allosteric communication theory that the free energies of a proportion of residue–residue interactions within the allosteric sites undergo considerable changes due to orthosteric perturbations. Hence, the significant differences of energetic decomposition at each residue pair serve as predictors of potential allosteric sites. Following this methodology, we first divided all residue pairs into three categories according to their interaction energy differences before and after orthosteric loading: small, medium, and large energy differences. The energy coupling score was defined as the ratio of residue pairs with large energy differences to those with medium energy differences, and a cutoff score was set at 0.25 to identify potential allosteric sites [69]. Namely, pockets with an energy coupling score over 0.25 were considered allosteric sites as they exhibited significant correlations with the orthosteric site. Herein, we first tested it with the MEK-pocket and obtained an energy coupling score of 0.4197, demonstrating the reliability of this approach (Figure 9E). It was further performed for the newly detected X-pocket, which gave an energy coupling score of 0.3710, far in excess of the threshold at 0.25 (Figure 9F). This confirmed that X-pocket was closely coupled with the orthosteric site through energetics, and it was regulated by orthosteric inhibitor binding via reversed allosteric communication. In addition, the residue pairs distributed in the third category presented allosteric residues with a remarkable correlation with orthosteric loading (Table S1). Together with the information of key residues composing the X-pocket given by Fpocket, the residues that displayed a combination of structural and energetic significance for pocket formation were identified as hot spots in the X-pocket, including K867, E868, H893, and K960. These hot spots not only were easily reached by small molecules because of their exposure to the pocket surface, but also possessed the potential to transmit allosteric information towards the orthosteric site. Owing to their pivotal roles in the emergence of the X-pocket, these hot spots guide further structural analysis and provide promising targets for future drug design. Together with the volume analysis, it concurred with our suggestion that X-pocket represented a novel, potential allosteric site that was highly correlated with the ATP-binding site, which served as a promising target for dual-targeting.

We further investigated the detailed composition of these allosteric pockets to represent more structural information for future drug design. As discussed above, the formation of the K745-E762 salt bridge mediated the inward movement of the αC-helix, and not only limited the volume of the MEK-pocket, but also increased the inner steric hindrance caused by the stack of residues (Figure 8A). On the other hand, the X-pocket was found deepened by an extended A loop, especially with K867 and E868 pointing to the more stable αF, αH, and αI helices (Figure 8B). Furthermore, the A loop is the only flexible component of the potential allosteric pocket, thus a decent X-pocket also exists in the activation state of EGFR, presenting an appealing target since it is the dominating conformation for the oncogenic mutants. The binding of small ligands at X-pocket can efficiently engender a mobility restriction of the A loop, which could impede substrate binding. As the X-pocket has never been reported in previous EGFR structures, our findings proposed that it may be a potential allosteric site for future drug design, especially in dual-targeting therapeutics.

### 3.6. The Nonconservative Potential Allosteric Pocket Enables Specific Targeting of EGFR across the Human Kinome

**Figure 10:**
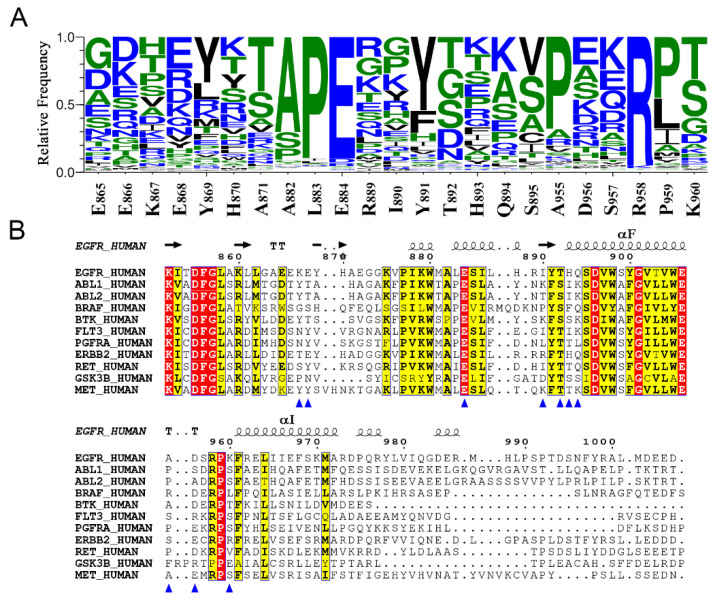
Sequence conservation of the allosteric pockets across the human kinome. (**A**) A sequence logo made by WebLogo3 server (http://weblogo.threeplusone.com/, accessed on 17 March 2021) based on sequence alignment of 518 kinases across the human kinome. The overall height of symbols within a stack indicates the relative frequency of each amino acid at the corresponding position. While the color indicates the hydrophobicity of the amino acid, with hydrophilic amino acids colored in blue, neutral ones in green, and hydrophobic ones in black. (**B**) Sequence alignment of EGFR, ABL1, ABL2, BRAF, BTK, FLT3, PGFR, ERBB2, RET, GSK3, and MET, representing the most important anti-cancer target in the human kinome. The residues forming the X-pocket are pointed to by the blue triangles.

Despite their various sequences, the approximately 518 kinases identified across the human kinome feature remarkable structural similarity with a characteristic fold. The X-pocket is mainly constituted by the activation segment, αF, αH, and αI helices, all of which are functionally and structurally conserved among various kinases, therefore a further sequence conservation analysis was carried out to discuss the potential of specific targeting of EGFR at the novel allosteric site. Herein, we first performed a global alignment of the human kinome and calculated the relative frequency of different amino acids within the X-pocket (Figure 10A). Although the Ala-Pro-Glu (APE) motif in the C-terminus of the A loop and the Arg in the αH-αI loop were highly conserved, most extracted residues in the A loop and the helices were less conserved. Focusing on the sequence of EGFR, the amino acids in 13 out of 23 extracted residues account for less than 10% of all aligned residues across the human kinome (E865, E866, K867, H870, A871, L883, I890, H893, Q894, A955, D956, S957, and K960), including 3 out of the 4 hot spots (K867, H893, and K960) in the X-pocket. Hence, these least conserved hot spots rendered potential allosteric targets with promising affinity, potency, and selectivity. Next, we explored the polarity of the X-pocket. Only 9 out of 23 residues were conserved in polarity in more than 75% of the human kinome, providing additional evidence for selective targeting. Moreover, we analyzed the sequence alignment of 11 clinically frequent oncogenic kinases, encompassing EGFR, ABL1, ABL2, BRAF, BTK, FLT3, PGFR, ERBB2, RET, GSK3, and MET (Figure 10B and Figure S7). We observed that most residues forming the X-pocket in EGFR were nonconservative, while K867, S895, and K960 were only present in EGFR, thereby offering the opportunity to utilize these residues to achieve selectivity among critical anti-cancer kinase targets. Since hot spots K867 and K960 were relatively tractable for salt bridge formation with small molecules, we suggested them as starting points for ligand design. These results validated the selectivity potential of the X-pocket, which indicated desirable efficacy for allosteric drugging to cooperate with orthosteric inhibitors.

## 4. Discussion

Here, we illuminated a dual-targeting therapeutic mechanism that orthosteric loading regulated allosteric sites via reversed allosteric communication, thereby exerting synergetic effects on improving the general efficacy. Dual-targeting with osimertinib and JBJ in EGFR^L858R/T790M^ was utilized to validate our proposal. We provided atomistic insight into the reversed allosteric communication and the subsequent conformational ensemble alterations induced by osimertinib binding at the orthosteric site. In particular, osimertinib transmitted the reversed signals to allosteric sites via a communication hub in the interlobular junction region, which triggered coordinative motions in different subdomains, thus stabilizing an intermediate state with a unique αC-in, A loop open, and DFG-out conformation. Significantly, a novel potential allosteric pocket (X-pocket) was identified, which was well exposed and can be readily targeted in both the active and intermediate states of EGFR. The coupling between the ATP-binding site and the X-pocket was validated using both structural and energetic dynamics computations, and further sequence conservation analysis revealed a non-conservation characteristic of the X-pocket across the human kinome, manifesting its potential to yield selective allosteric inhibitors targeting EGFR. Our work provided a mechanistic basis for future clinical application of combining orthosteric and allosteric drugs, and presented a novel avenue to detect allosteric sites by orthosteric perturbations.

In the era of modern biomedicine, drug resistance remains a clinically insuperable obstacle, which poses a leading challenge especially in the treatment of infection and cancers, thus threatening global public health [70,71,72,73]. In the last decades, the long-term impetus has promoted innovative attempts to tackle the elusive enigma with multidisciplinary approaches. With accumulating evidence of the multiple advantages boosted by allosteric modulation, it has attracted an ever-growing interest in pharmaceutics, and consequently, the combination of orthosteric and allosteric agents is emerging as a revolutionary strategy to overcome resistance, as exemplified by the growing number of successful cases [74,75,76,77,78,79]. Accordingly, a series of orthosteric-allosteric drug combinations have proceeded into clinical trials, mostly involving oncology, viruses, and autoimmune disorders [17]. In addition to BCR-ABL and EGFR, another quintessential example is targeting nonstructural protein 5A (NS5A) for the treatment of hepatitis C virus (HCV). To overcome the drug-resistance against the first-class orthosteric inhibitor daclatasvir, an allosteric synergist Syn-395 was developed [21]. Additional loading of Syn-395 altered the overall structure of NS5A daclatasvir complex, leading to a 1000-fold enhancement in the potency of the orthosteric inhibitor. Another recent success was achieved in selectively inhibiting plasmodium falciparum hexose transporter 1 (PfHT1) with orthosteric-allosteric dual inhibitors for anti-malarial treatment [18]. Huang et al. designed a set of small-molecules that simultaneously block the orthosteric and allosteric pockets of PfHT1, constituting a novel weapon against multidrug-resistant malaria parasites. It was previously reviewed that in dual-targeting therapeutics, allosteric regulators bind to the recalcitrant target and precisely resensitize the orthosteric site in two major pathways: either through microcosmically reshaping the intrinsic residue network within the protein, or macroscopically shifting the conformational ensemble towards a favorable state [17]. In fact, these abovementioned resensitizing effects discovered in multiple cases imply the traditional allosteric communication pattern where signals propagate from the allosteric site to the orthosteric site. However, an extra improvement in the affinity of allosteric agents during the dual-targeting therapeutics was further reported, which manifested a reversed signaling pathway within the target. Recently, a bidirectional model of allosteric signaling has been proposed and validated in several previous reports [23,24,25,26]. Moreover, recent success in the development of a single molecule spanning both the ATP binding site and the allosteric sites also support an intrinsic connection between the topologically distant sites [80,81].

In our study, we revealed a reversed allosteric communication mechanism underlying the unique phenomenon found in dual-targeting, which indicates that the proper combination of orthosteric and allosteric drugs has the potential to not only circumvent drug-resistance, but also synergistically improve the efficacy of both therapeutic agents. Hence, our results provide theoretical basis for the dual-targeting therapeutics to become a universally standard practice.

Despite the recent advances in allosteric regulation, the effective allosteric modulators remain relatively rare in comparison with traditional orthosteric drugs, indicating tougher challenges faced by this emerging field. The detection of allosteric sites, a pre-requisite for allosteric drug development, represents a major obstacle. Historically, allosteric site prediction was once based on serendipitous discovery, while the latest decade has witnessed the upsurge of computational tools aiding the structure-based rational design of allosteric regulators [82,83,84,85,86,87,88]. Due to a constantly deepening understanding of protein allostery, and the rapid development of diverse bioinformatics methodologies, the characterization and identification of potential allosteric sites was considerably facilitated. However, the present computation models and algorithms pose non-negligible restrictions to the application of these computational aids. First, because of the relatively elusive understanding of protein allostery, algorithm development is faced with great challenges to accurately approximate and reveal allosteric effects. Second, the nonphysical perturbations, such as dummy atoms and artificial mutations, applied for allosteric signal analysis notably hinder the performance of the prediction methods. Thirdly, current knowledge of protein allosteric regulation is mainly concentrated on a certain list of protein classes, represented by kinases and GPCRs. Consequently, the performance of data-based prediction methods is inevitably hampered by a biased dataset for model training and optimization. Moreover, current efforts to computationally predict allosteric sites are based on the experimentally revealed crystal structures and focus only on the pockets present in the existing structures, while ignoring the vast potential of massive cryptic allosteric sites that only emerge during conformational dynamics within the target. Last, but not least, in terms of dual-targeting therapeutics, some allosteric sites may overlap with the orthosteric sites, thus impeding the utility. In our study, we developed an approach to identify potential allosteric sites tailored for dual-targeting therapeutics. Based on a dynamic ensemble of the protein target, the reversed allosteric communication triggered by orthosteric drugging was exploited as the mechanistic starting point to detect allosteric sites. Compared with the previous computational methods, we circumvented the obstacles of biased databases and imperfect prediction algorithms. MD simulations of a protein target in complex with its orthosteric agent enabled the discovery of cryptic allosteric sites in a state resembling the physiological conditions. Importantly, non-overlapping allosteric pockets can be intuitively selected to ensure the practicality of dual-targeting therapeutics. Such unbiased detection methods can expand the spectrum of available allosteric pockets, facilitate the conceptualization of novel, potent, and selective kinase inhibitors, hopefully paving an avenue towards the application of combining orthosteric and allosteric drugs.

Major limitations include that the conformational landscape of EGFR was not fully sampled during our simulations since the relative timescale for completed conformational shifts reached hundreds of microseconds [8]. In addition, we only focused on the intracellular CD, leaving the extracellular receptor and the transmembrane region unexplored. Although our study revealed a potential allosteric pocket in EGFR, further experimental validation and lead compound identification are needed for allosteric drug design. Future studies are expected to provide a more comprehensive understanding of the orthosteric ligand fine-tuning EGFR and to realize X-pocket as a versatile allosteric pocket in human kinases.

## 5. Conclusions

Here, we untangled an underlying mechanism of reversed allosteric communication in dual-targeting therapeutics, by which the allosteric sites were regulated by orthosteric drugs. The reliability of this proposal was validated by the correlation between the previously identified allosteric MEK-pocket in EGFR and the orthosteric ATP-binding site. A novel, potential allosteric site was identified in EGFR based on its remarkable coupling with the orthosteric site, both structurally and energetically. Our results laid a theoretical foundation for the development of the dual-targeting strategy in massive therapeutic targets. Moreover, X-pocket, the non-overlapping allosteric pocket we detected in EGFR mutants, provided a potential alternative to yield selective allosteric agents, which can synergistically function with the traditional TKIs. X-pocket is mostly composed of nonconservative residues, including hot spots K867, S895, and K960. Therefore, X-pocket renders potential targets with promising affinity, potency, and selectivity for future allosteric drug design and the dual-targeting strategy.

## Data Availability

The crystal structures of human EGFR^L858R/T790M^ monomeric form (PDB ID: 4I1Z) and EGFR^L858R/T790M^–osimertinib–JBJ (PDB ID: 6DUK) were downloaded from the Protein Data Bank.

## Supplementary Materials

The following are available online at https://www.mdpi.com/article/10.3390/pharmaceutics13050747/s1, Figure S1: Starting structures of the EGFR^L858R/T790M^, Figure S2: Root-mean-square deviations (RMSD) of Cα atoms in the EGFR^L858R/T790M^, EGFR^L858R/T790M^–osimertinib, and EGFR^L858R/T790M^–osimertinib–JBJ systems, Figure S3: RMSF and RMSD differences, Figure S4: PCA analysis, Figure S5: The representative structures of the secondary free energy minima, Figure S6: Pocket characterization, Figure S7: Sequence alignment of EGFR, ABL1, ABL2, BRAF, BTK, FLT3, PGFR, ERBB2, RET, GSK3, and MET, Table S1: Residue-residue interactions in the X pocket with medium or large energy differences.
