# Supplementary material for: Untangling Dual-Targeting Therapeutic Mechanism of Epidermal Growth Factor Receptor (EGFR) Based on Reversed Allosteric Communication
Source: Pharmaceutics. 2021 May 18;13(5):747. doi: 10.3390/pharmaceutics13050747 (PMC8158526; doi:10.3390/pharmaceutics13050747)
Supplement: Supplementary file 1 [file pharmaceutics-13-00747-s001.zip › pharmaceutics-1174079-supplementary.pdf]

pharmaceutics

Supplementary Materials: Untangling Dual-Targeting Therapeutic Mechanism based on Reverse Allosteric Communication

Yuran Qiu, Xiao-Lan Yin, Xinyi Li, Yuanhao Wang, Qiang Fu, Renhua Huang and Shaoyong Lu

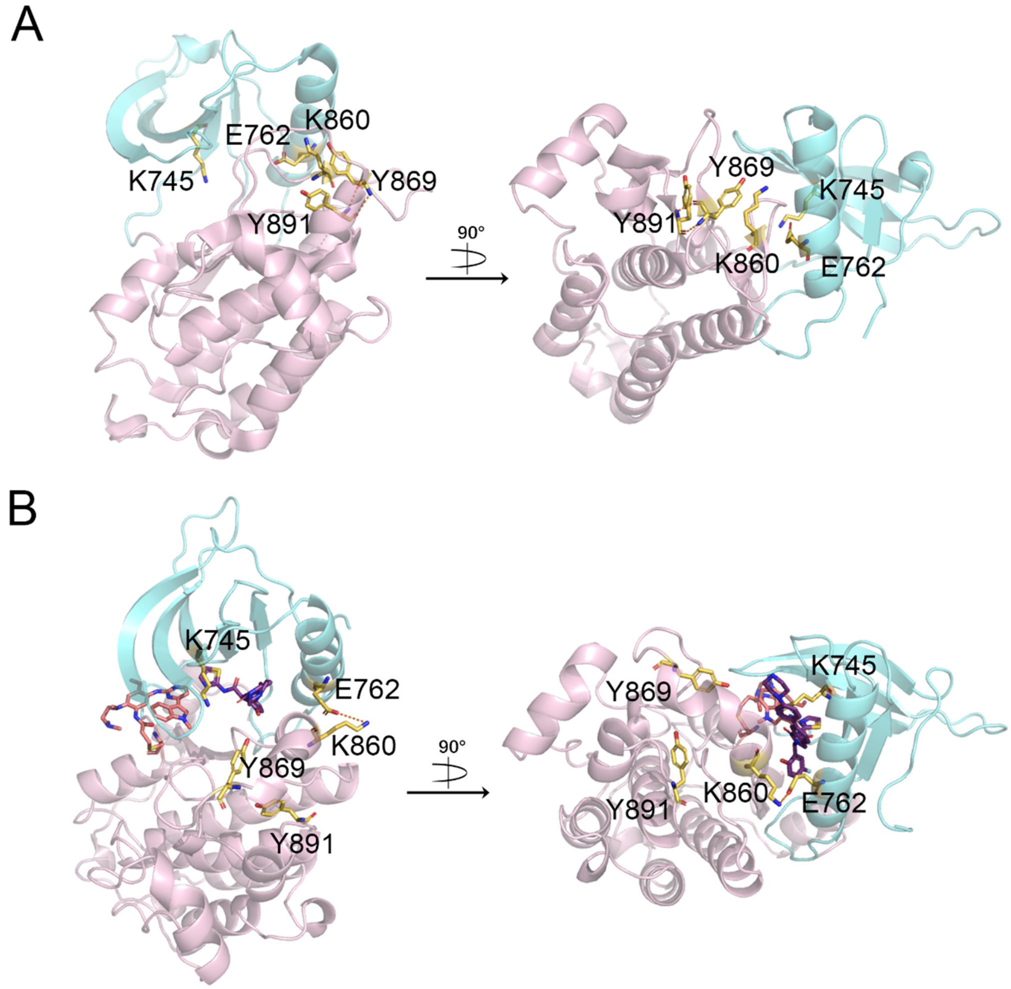

Figure S1. Starting structures of the EGFR$^{L858R/T790M}$ **(A)** and EGFR$^{L858R/T790M}$–osimertinib–JBJ-04-125-02 **(B)** systems. N-lobe and C-lobe are colored in cyan and pink, respectively. Key residues are depicted by yellow sticks and the salt bridges are depicted by orange dashed lines.

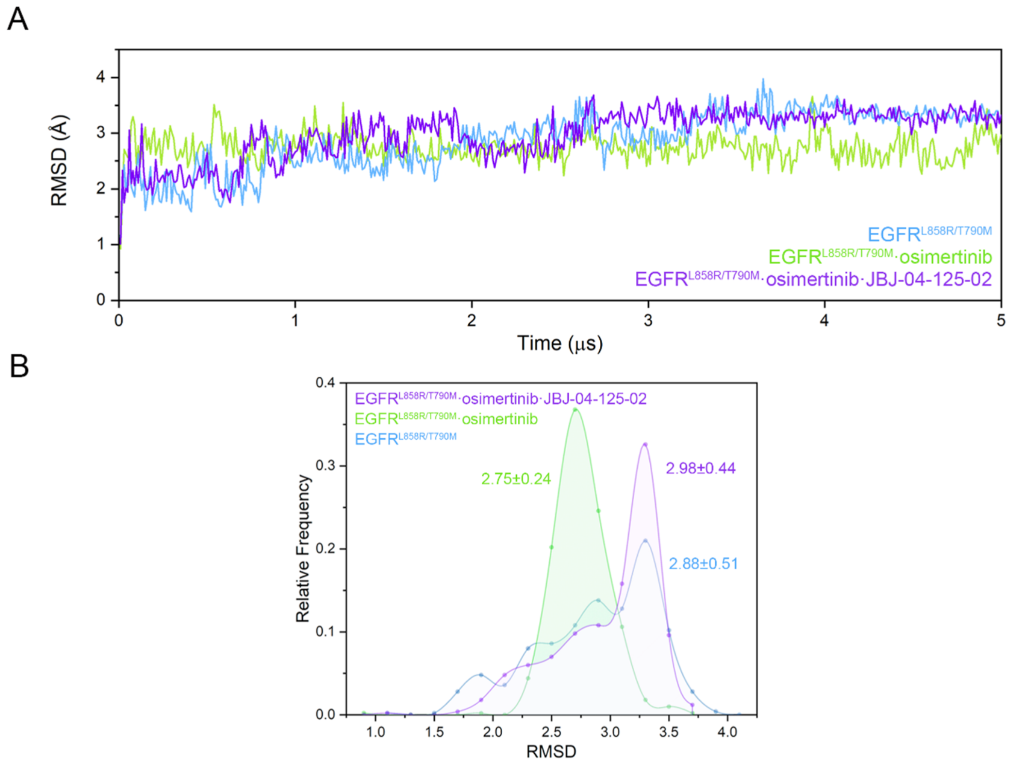

Figure S2. Root-mean-square deviations (RMSD) of Cα atoms in the $EGFR^{L858R/T790M}$ (blue), $EGFR^{L858R/T790M}$–osimertinib (green), and $EGFR^{L858R/T790M}$–osimertinib–JBJ-04-125-02 systems (purple) depicted in the curve chart **(A)** and the frequency distribution graph **(B)**.

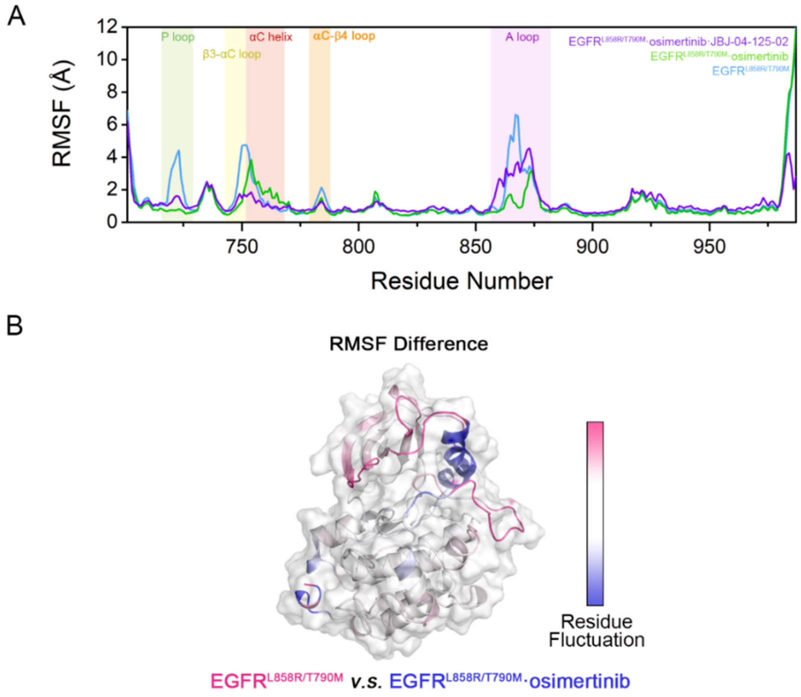

Figure S3. (A) Root-mean-square fluctuations (RMSF) of Cα atoms in the $EGFR^{L858R/T790M}$ (blue), $EGFR^{L858R/T790M}$–osimertinib (green), and $EGFR^{L858R/T790M}$–osimertinib–JBJ-04-125-02 systems (purple). Major functional regions with significant differences among systems were highlighted by green (P loop), yellow (β3-αC loop), red (αC helix), orange (αC-β4 loop), and purple (A loop) background, respectively. **(B)** RMSD difference between the $EGFR^{L858R/T790M}$ and the $EGFR^{L858R/T790M}$–osimertinib system (RMSF difference = $RMSF_{apo}$–$RMSF_{holo}$) was projected onto the structure of EGFR, in which the pink and

blue region reflected the more fluctuations within the EGFR$^{L858R/T790M}$ and the EGFR$^{L858R/T790M}$–osimertinib system, respectively.

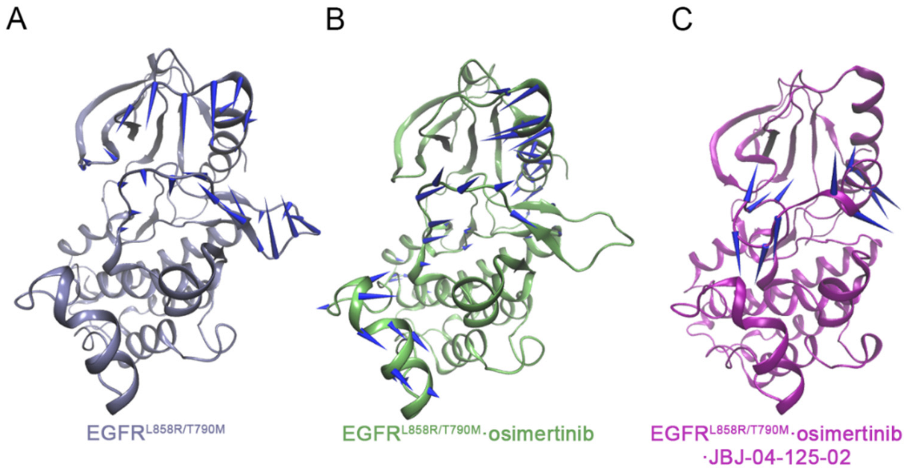

Figure S4. PCA analysis of **(A)** EGFR$^{L858R/T790M}$ system, **(B)** EGFR$^{L858R/T790M}$–osimertinib system, and **(C)** EGFR$^{L858R/T790M}$–osimertinib–JBJ-04-125-02 system. The porcupine plots were drawn with VMD to visualize the major movements along with PC1 obtained from PCA, with blue arrows depicted the directions of protein motions, whereas the length of the arrows represented the magnitude of the movements.

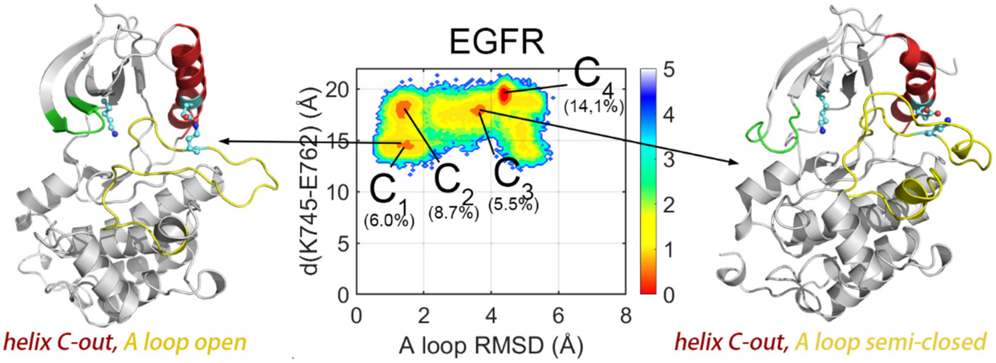

Figure S5. The representative structures of the secondary free energy minima are shown with the αC helix colored in red, the K745-E762 residue pair in blue, the A loop in yellow, and the P loop in green. The unit of free-energy values is kcal/mol.

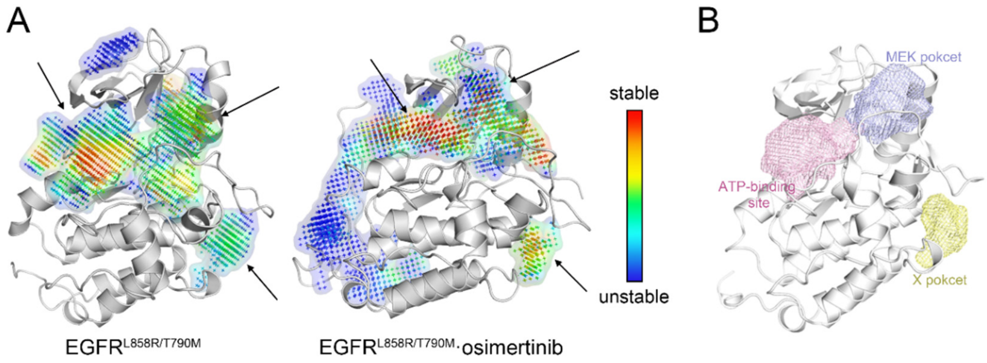

Figure S6. (A) Stability of pockets in the EGFR $^{L858R/T790M}$ (left) and the EGFR $^{L858R/T790M}$–osimertinib (right) systems. **(B)** Overview of the ATP-binding site (pink), the MEK-pocket (blue), and the X-pocket (yellow).

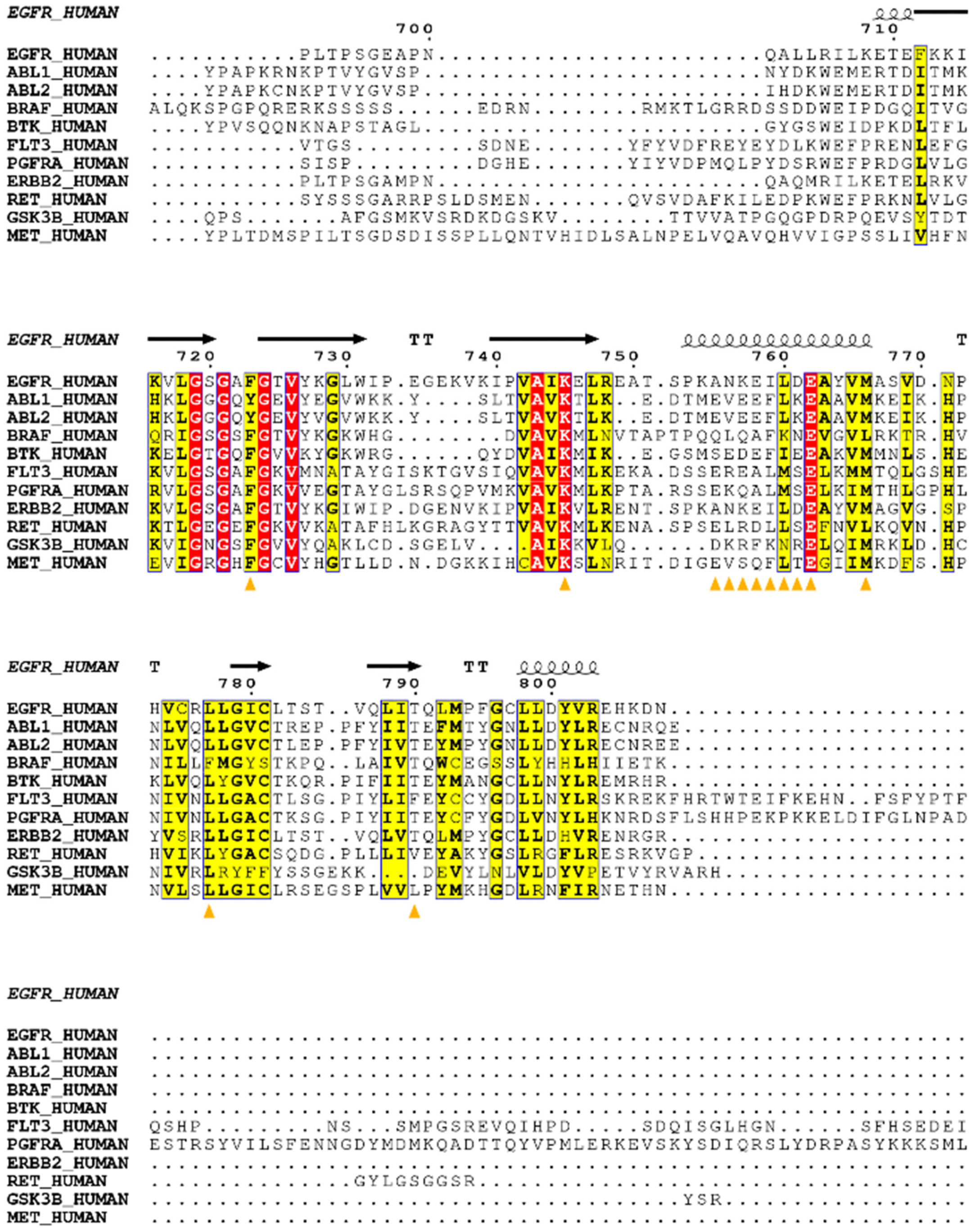
EGFR_HUMAN
700
710
EGFR_HUMAN ....PLTPSGEAPN.......................QALLRILKETEFKKI
ABL1_HUMAN ....YPAPKRNKPTVYGVSP........................NYDKWEMERTDITMK
ABL2_HUMAN ....YPAPKCNKPTVYGVSP........................IHDKWEMERTDITMK
BRAF_HUMAN ALQKSPGPQRERKSSSSS......EDRN........RMKTLGRRDSSDDWEIPDGQITVG
BTK_HUMAN ....YPVSQQNKNAPSTAGL........................GYGSWEIDPKDLTFL
FLT3_HUMAN ...........VTGS.........SDNE.......YFYVDFREYEYDLKWEFPRENLEFG
PGFRA_HUMAN ...........SISP.........DGHE.......YIYVDPMQLPYDSRWEFPRDGLVLG
ERBB2_HUMAN ....PLTPSGAMPN.......................QAQMRILKETELRKV
RET_HUMAN ...........SYSSSGARRPSLDSMEN.......QVSVDAFKILEDPKWEFPRKNLVLG
GSK3B_HUMAN ....QPS........AFGSMKVSRDKDGSKV........TTVVATPGQGPDRPQEVSYTDT
MET_HUMAN ....YPLTDMSPILTSGDSDISSPLLQNTVHIDLSALNPELVQAVQHVVIGPSSLIVHFN
EGFR_HUMAN
TT
T
720
730
740
750
760
770
EGFR_HUMAN KVLGSGAFGTVYKGLWIP.EGEKVKIPVAIKELREAT.SPKANKEILDEAYVMASVD.NP
ABL1_HUMAN HKLGGGQYGEVYEGVWKK.Y....SLTVAVKTLK..E.DTMEVEEFLKEAAVMKEIK.HP
ABL2_HUMAN HKLGGGQYGEVYVGVWKK.Y....SLTVAVKTLK..E.DTMEVEEFLKEAAVMKEIK.HP
BRAF_HUMAN QRIGSGSFGTVYKGKWHG........DVAVKMLNVTAPTPQQLQAFKNEVGVLRKTR.HV
BTK_HUMAN KELGTGQFGVVKYGKWRG......QYDVAIKMIK..E.GSMSEDEFIEEAKVMMNLS.HE
FLT3_HUMAN KVLGSGAFGKVMNATAYGISKTGVSIQVAVKMLKEKA.DSSEREALMSELKMMTQLGSHE
PGFRA_HUMAN RVLGSGAFGKVVEGTAYGLSRSQPVMKVAVKMLKPTA.RSSEKQALMSELKIMTHLGPHL
ERBB2_HUMAN KVLGSGAFGTVYKGIWIP.DGENVKIPVAIKVLRENT.SPKANKEILDEAYVMAGVG.SP
RET_HUMAN KTLGEGEFGKVVKATAFHLKGRAGYTTVAVKMLKENA.SPSELRDLLSEFNVLKQVN.HP
GSK3B_HUMAN KVIGNGSFGVVYQAKLCD.SGELV....AIKKVLQ......DKRFKNRELQIMRKLD.HC
MET_HUMAN EVIGRGHFGCVYHGTLLD.N.DGKKIHCAVKSLNRIT.DIGEVSQFLTEGIIMKDFS.HP
EGFR_HUMAN
T
TT
780
790
800
EGFR_HUMAN HVCRLLGICLTST..VQLITQLMPFGCLLDYVREHKDN.....................
ABL1_HUMAN NLVQLLGVCTREP.PFYIITEFMTYGNLLDYLRECNRQE....................
ABL2_HUMAN NLVQLLGVCTLEP.PFYIVTEYMPYGNLLDYLRECNREE....................
BRAF_HUMAN NILLFMGYSTKPQ..LAIVTQWCEGSSLYHHLHIIETK.....................
BTK_HUMAN KLVQLYGVCTKQR.PIFIITEYMANGCLLNYLREMRHR.....................
FLT3_HUMAN NIVNLLGACTLSG.PIYLIFEYCCYGDLLNYLRSKREKFHRTWTEIFKEHN..FSFYPTF
PGFRA_HUMAN NIVNLLGACTKSG.PIYIITEYCFYGDLVNYLHKNRDSFLSHHPEKPKKELDIFGLNPAD
ERBB2_HUMAN YVSRLLGICLTST..VQLVTQLMPYGCLLDHVRENRGR.....................
RET_HUMAN HVIKLYGACSQDG.PLLLIVEYAKYGSLRGFLRESRKVGP...................
GSK3B_HUMAN NIVRLRYFFYSSGEKK....DEVYLNLVLDYVPETVYRVARH.................
MET_HUMAN NVLSLLGICLRSEGSPLVVLPYMKHGDLRNFIRNETHN.....................
EGFR_HUMAN
EGFR_HUMAN ....
ABL1_HUMAN ....
ABL2_HUMAN ....
BRAF_HUMAN ....
BTK_HUMAN ....
FLT3_HUMAN QSHP.........NS...SMPGSREVQIHPD.....SDQISGLHGN......SFHSEDEI
PGFRA_HUMAN ESTRSYVILSFENNGDYMDMKQADTTQYVPMLERKEVSKYSDIQRSLYDRPASYKKKSML
ERBB2_HUMAN ....
RET_HUMAN ....GYLGSGGSR....................................
GSK3B_HUMAN ....YSR...........
MET_HUMAN ....

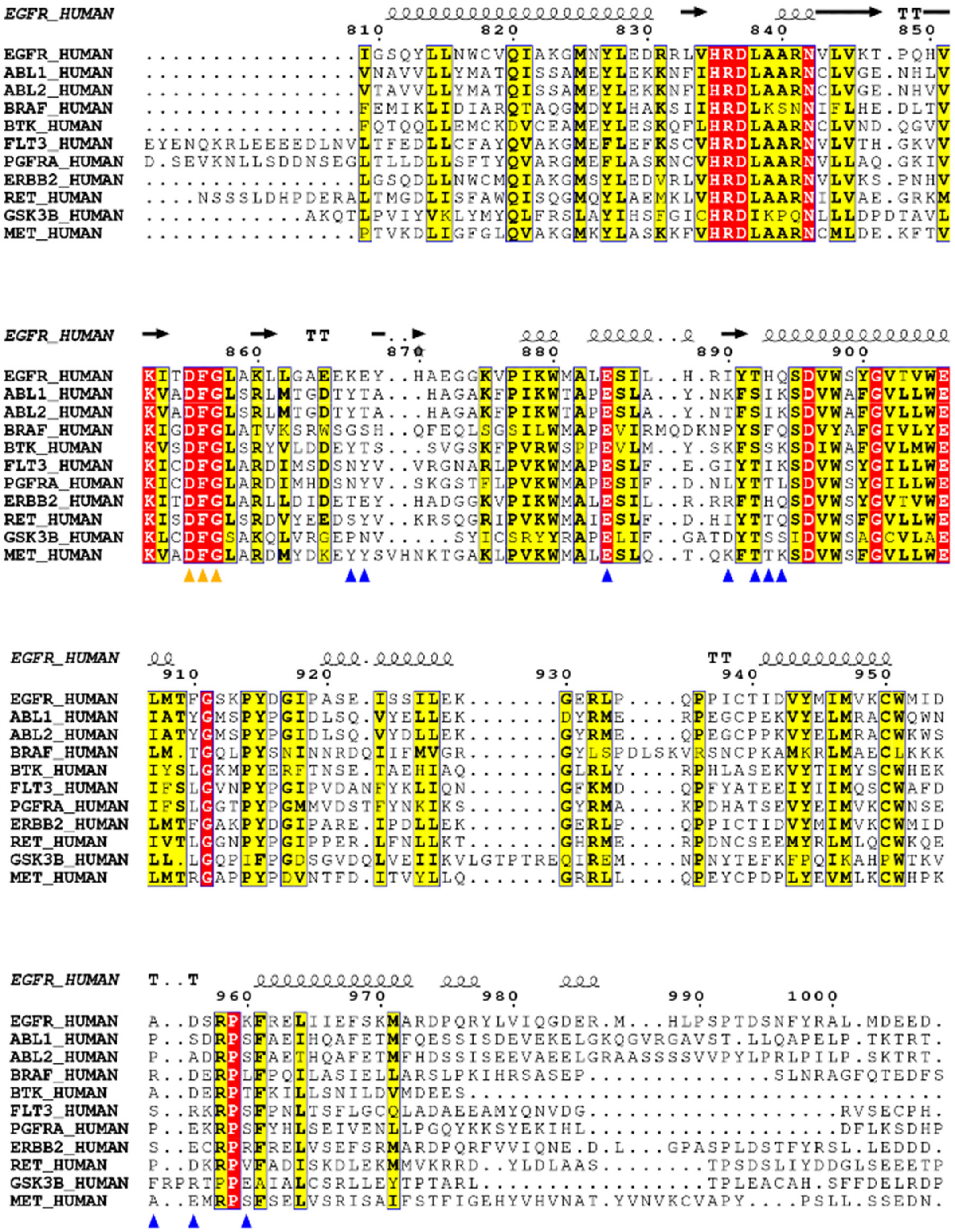

Figure S7. Sequence alignment of EGFR, ABL1, ABL2, BRAF, BTK, FLT3, PGFR, ERBB2, RET, GSK3, and MET. The residues forming the X-pocket and the MEK-pocket are pointed to by the blue and orange triangles, respectively.

Table S1. Residue-residue interactions in the X pocket with medium or large energy differences[a.]

	Residue 1	Residue 2	E_{apo}	E_{holo}	ΔE	Std. Dev. $_{apo}$	Std. Dev. $_{holo}$
1	MET 825	HIE 835	-0.9573	-2.1845	1.2272	0.1974	1.2101
2	MET 825	HIE 893	-0.5338	-0.1669	-0.3669	0.3439	0.0738
3	MET 825	PHE 961	-3.7551	-3.5754	-0.1798	0.3245	0.3877
4	ASN 826	PHE 961	-1.8497	-2.1759	0.3262	0.6133	0.4914
5	GLU 829	LEU 833	-0.3273	-0.6605	0.3332	0.2490	0.3123
6	GLU 829	HIE 893	-2.9778	-2.6696	-0.3082	1.0807	0.6015
7	GLU 829	LYS 960	-2.6584	-1.9097	-0.7488	4.0318	3.2972
8	GLU 829	PHE 961	-1.1195	-1.5502	0.4307	0.4419	0.4398
9	GLU 829	ARG 962	-3.0614	-2.3352	-0.7261	4.1218	3.3502
10	ARG 832	LEU 862	-4.0452	-4.6113	0.5661	1.3760	1.1919
11	ARG 832	GLU 865	-0.2294	-0.4793	0.2499	1.0484	0.8529
12	ARG 832	GLU 866	-0.7301	-3.4147	2.6846	1.6229	2.5436
13	ARG 832	LYS 867	-0.1032	-0.4682	0.3650	0.3057	0.3993
14	ARG 832	HIE 893	-0.4036	-1.7168	1.3132	0.5123	1.1650
15	LEU 833	HIE 893	-1.8071	-1.4675	-0.3396	1.0861	0.5166
16	LEU 833	ASP 896	-0.6648	-0.8733	0.2085	0.1896	0.2987
17	VAL 834	TYR 869	-0.3210	-0.9654	0.6444	0.3981	0.3205
18	VAL 834	HIE 893	-2.5650	-1.9398	-0.6252	1.0015	0.5078
19	VAL 834	ASP 896	-6.1670	-6.7851	0.6181	0.8414	0.8956
20	HIE 835	ASP 896	-5.9740	-10.6266	4.6526	0.9086	2.8292
21	HIE 835	PHE 961	-0.0630	-0.2372	0.1742	0.0189	0.1512
22	LEU 862	GLU 866	-0.7682	-2.3728	1.6046	1.1177	1.2625
23	LEU 862	LYS 867	-0.4125	-1.0923	0.6798	0.6072	0.3620
24	LEU 862	GLU 868	-0.3067	-1.2420	0.9353	0.5729	0.3049
25	LEU 862	TYR 869	-0.5817	-1.2485	0.6668	0.7714	0.2939
26	LEU 862	TYR 891	-0.4189	-0.2239	-0.1949	0.3244	0.1010
27	LEU 862	THR 892	-0.6610	-0.1977	-0.4633	0.4007	0.1917
28	LEU 862	HIE 893	-1.3190	-1.1309	-0.1881	0.5465	0.2809
29	GLU 865	TYR 869	-0.7906	-0.0171	-0.7735	1.1794	0.0057
30	GLU 865	HIE 870	-0.2972	-0.0069	-0.2903	0.7620	0.0053
31	GLU 865	ARG 889	-0.4343	-0.0096	-0.4247	1.1136	0.0035
32	GLU 865	ILE 890	-0.1755	-0.0014	-0.1742	0.4293	0.0018
33	GLU 865	TYR 891	-0.1969	0.0011	-0.1980	0.4206	0.0016
34	GLU 866	HIE 870	-0.4586	-0.0914	-0.3672	0.8205	0.2175
35	LYS 867	ARG 889	-0.2752	0.0364	-0.3116	0.9071	0.0169
36	LYS 867	ILE 890	-0.1587	0.0832	-0.2419	0.4919	0.0580
37	LYS 867	THR 892	-0.4240	-1.1678	0.7439	0.8157	0.9184
38	LYS 867	HIE 893	-0.0737	-0.2560	0.1823	0.1882	0.3512
39	GLU 868	ILE 890	-0.3350	-0.9615	0.6265	0.5573	0.3879
40	GLU 868	TYR 891	-0.4867	-2.2697	1.7830	0.9542	0.5422
41	GLU 868	THR 892	-0.3030	-1.1068	0.8038	0.5638	0.3764
42	TYR 869	ILE 890	-0.8223	-2.4212	1.5989	1.1506	0.4861
43	TYR 869	TYR 891	-1.9199	-4.7069	2.7870	2.1241	0.6538
44	TYR 869	THR 892	-0.1734	-0.3463	0.1729	0.2195	0.0930
45	HIE 870	HIE 888	-0.3422	-0.8043	0.4622	0.4161	0.8402
46	HIE 870	ILE 890	-0.5222	-1.7968	1.2746	0.7529	0.6236
47	HIE 870	TYR 891	-0.4761	-0.7498	0.2737	0.5647	0.2920
48	LEU 883	TRP 898	-2.4874	-2.0479	-0.4395	0.5295	0.6873
49	GLU 884	ARG 889	-1.2523	-0.9885	-0.2639	0.5441	0.4838
50	GLU 884	ILE 890	-4.5502	-4.1662	-0.3840	0.6977	0.9122
51	GLU 884	SER 895	-4.4255	-4.1586	-0.2669	1.9253	1.5507
52	GLU 884	ALA 955	-0.8663	-0.6053	-0.2611	0.3539	0.3319
53	SER 885	ARG 889	-2.5563	-1.5640	-0.9923	1.7614	1.0036
54	TYR 891	SER 895	-1.4311	-1.6822	0.2511	0.5298	0.4940
55	HIE 893	VAL 897	-1.7232	-1.5168	-0.2064	0.6041	0.5919
56	GLN 894	ASP 956	-1.7398	-1.3318	-0.4080	1.3943	0.8669
57	GLN 894	ARG 958	-4.8253	-4.6602	-0.1651	1.1817	1.2857

58	SER 895	ARG 958	-0.7921	-0.9663	0.1742	0.5329	0.5387
59	VAL 897	PHE 961	-2.5021	-2.3061	-0.1960	0.3485	0.3440
60	MET 952	ARG 958	-8.2599	-7.8620	-0.3978	1.0434	1.0804
61	ILE 953	ARG 958	-2.3903	-2.6922	0.3019	1.0210	1.0897
62	ASP 956	LYS 960	-0.9931	-0.7678	-0.2253	2.0096	1.5434

a. Residue-residue interactions with large energy differences are highlighted in green background.
